# The gender wealth gap and the role of private pension wealth in Great Britain

**DOI:** 10.1007/s11150-026-09840-6

**Published:** 2026-03-13

**Authors:** Brian Nolan, Juan C. Palomino

**Affiliations:** 1 Department of Social Policy and Intervention, Institute for New Economic Thinking, and Nuffield College, University of Oxford, Oxford, UK; 2 Universidad Complutense de Madrid and Institute for New Economic Thinking, Oxford, UK

**Keywords:** Wealth, Gender gap, Pension, D31, D63, D64

## Abstract

Research on gender wealth gaps is hampered by the fact that most wealth surveys gather information on wealth at the household or family level rather than for individuals, and also often do not include pension wealth. Here we exploit the rich data on individual wealth including private pension wealth (occupational and personal) obtained by the British Wealth and Assets Survey (WAS) to assess the gender wealth gap in Great Britain between 2006–08 and 2018–20, with a particular focus on the prominent role of private pension wealth. We find that in 2018–20 mean wealth for women was 34.0% lower than for men when pension wealth was included versus only 16.3% lower when it was not. Decomposition analysis shows that differences between men’s and women’s observed socioeconomic characteristics account for a significant share of the private pension wealth gap, with differences in labour market experiences (labour income, career gaps and part-time jobs) playing a key role. We also find an increase in the absolute gender pension gap over time that is associated with the rising proportion of pension wealth held in Defined Contribution (DC) schemes that are based on the return on accumulated pension contribution ‘pots’ rather than Defined Benefit (DB) schemes where entitlement is based on salary and years of service. Financial and business wealth is also an important contributor to the overall wealth gap, and like pension wealth its contribution is seen to increase towards the top of the distribution.

## Introduction

Research on the distribution of wealth has burgeoned across the social sciences in recent years, fuelled by the growing availability of high-quality micro-data from household wealth surveys. Measures of wealth inequality across the distribution in terms of indices or wealth shares show it to be much greater than inequality in the income distribution. This has major implications for differences across individuals and households in welfare and opportunities. Having a wealth ‘buffer’ is a key source of security and well-being, regardless of the income level (Headey & Wooden, 2004; Hochman & Skopek, 2013; D’Ambrosio et al., 2020; Petrov & Romaguera-de-la-Cruz, 2025). Moreover, insufficient wealth buffers can prevent those at the lower end of the distribution from coping with an unexpected income drop, as was the case during the recent COVID-19 pandemic shock (Kuypers et al., 2022). From a poverty perspective, notably in old age, the combination of inadequate incomes and little or no assets is particularly problematic, as highlighted in various studies focused on ‘asset poverty’ or adopting a joint income-wealth perspective on capturing poverty empirically (see for example Haveman & Wolff, 2004, Brandolini et al., 2010, Azpitarte, 2012, Kuypers & Marx, 2018). Recently, Caminada et al., (2025) have found a strong link between pension benefits and changes in poverty among the elderly between 2005 and 2022 in 16 European countries. In terms of opportunities, wealth levels also serve as collateral for undertaking risky entrepreneurial projects or accessing credit markets.

The extent to which wealth differences occur also in the gender dimension is therefore of great relevance, since it reflects a source of disadvantage for women related but distinct from what occurs in the labour market and in the income flows. Thus, gender differences in wealth have indeed received increasing attention, (e.g. Deere & Doss, 2006; Sierminska, Frick, & Grabka 2010; Ruel & Hauser 2013; Grabka, Marcus, & Sierminska 2015; Lersch, Jacob, & Hank 2017; Schneebaum et al. 2018; Sierminska et al., 2018). However, in-depth investigation of gender wealth gaps and the underlying processes at work have been severely hampered by the fact that the most common practice in these surveys is to measure wealth at the household rather than individual level. Some studies (Ruel & Hauser 2013; Schneebaum et al. 2018) have dealt with this by comparing ‘female-headed’ households to ‘male-headed’ ones, but evidence from the few countries for which individual-level wealth data is available – notably Germany which has been studied relatively intensively (Sierminska et al., 2018; Kapelle & Lersch, 2020) - suggests that the gender wealth gap is actually larger among couple-headed households.

This highlights the importance of having individual-level wealth data in studying gender gaps, including the in-depth information required to estimate private pension wealth, and of fully exploiting such data where it is available. Here we take advantage of the fact that the Wealth and Asset Survey (WAS), the main official source of micro-level wealth data for Great Britain, has from its initiation in 2006–2008 sought to measure wealth (for the most part) at the individual level, and to include information that allows pension wealth to be incorporated. We employ the rich data provided by the WAS to assess the extent and nature of the gender wealth gap in Great Britain and how it has evolved since before the Great Recession, with a particular focus on the role of pension wealth.^1^

Furthermore, accumulated pension entitlements represent a very important form of command over resources in the future that play a central role for many individuals and households and are known to display marked gendered differences, but these are most often not included in studies of the gender wealth gap, primarily due to data limitations. These arise because the valuation of accumulated entitlements to future pensions involves application of complex actuarial formulae to detailed individual-level information about contributions and the nature of pension schemes and obtaining that information from survey respondents is particularly demanding.

The term “augmented wealth” is often used to refer to the broader definition of wealth that includes entitlements to future pension streams (Davies & Shorrocks, 2000), and this is seen in research on wealth inequality as a valuable complement to the more common focus on net worth (OECD, 2013). Outside the UK, national studies of the distribution of augmented wealth include Wolff, 2015 for the USA; Roine & Waldenström, 2009 for Sweden; Maunu, 2010 for Finland; Bönke, Grabka, Schröder, Wolff & Zyska, 2019 for Germany; Kuhn, 2020 for Switzerland. There have been fewer comparative studies but Cowell, Nolan, Olivera & Van Kerm, (2017) for example explore the distributional effects of including public and private pension wealth in an augmented measure of household wealth in 13 European countries participating in the first and available round (circa 2010) of the Household, Finance and Consumption Survey (HCFS). There have also been studies of UK wealth inequality that have sought to incorporate pension wealth, such as Crawford & Hood (2016), as well as official estimates from the Office for National Statistics (ONS) (ONS, 2019a, 2022).

In general, such studies find that the inclusion of pension wealth, especially from public pensions, reduces the degree of overall wealth inequality to an extent that varies across sources and countries. However, as one may expect given that pension rights reflect labour market differences even more than other assets, the implications for the gender wealth gap of using ‘augmented wealth’ - and in particular including private pensions - are different than for overall inequality, as we will see below.

Having discussed relevant previous research in Section 2, Section 3 describes the WAS data to be employed in our analysis. Section 4 sets out the main features of the gender wealth gap in Great Britain by family type and age, with and without private pension wealth. It also examines the gender wealth gap across the wealth distribution for different types of wealth, including pension wealth, and how that evolved through the Great Recession and recovery. Section 5 concentrates on private pension wealth, distinguishing by type of pension scheme in the working age population. In Section 6, we apply Oaxaca-Blinder decomposition to the gap between men and women in pension wealth, assessing the endowment and return effect of a range of variables, capturing labour market experiences but also the type of pension plan and attitudes to saving, risk and pensions. Finally, Section 7 brings together the main findings and highlights priorities for future research.

## Background

Gender wealth gaps are particularly difficult to capture empirically, even before trying to bring pension wealth into the picture. With wealth surveys most often seeking information on assets and debts only at household level, many studies on the topic have focused on single-headed households (Deere & Doss, 2006; Ruel & Hauser 2013; Schneebaum et al. 2018). Schneebaum et al. (2018) for example examined the gap in wealth between male and female single households using 2010 HFCS data for eight European countries while Ravazzini & Chesters (2018) compared data from national surveys for Australia and Switzerland on the same basis. Results for this sub-set of households may not however be representative of all households including couples. Using HFCS and machine learning techniques, Kukk et al., (2023) predict individual wealth from (mostly) household wealth data and individual characteristics including private pension wealth (defined contribution plans being available at the individual level); across the twenty-two European countries they find a mean gender gap ranging from 13% in Croatia and Hungary up to 42% in Luxembourg, or 24% on average.

The German Socio-Economic Panel (SOEP) is one of few surveys collecting wealth information at the individual level (in specific occasional modules) making intra-household comparisons possible, and this has been employed to directly analyse gender wealth gaps across both single-adult and couple households by for example Grabka et al. (2015), Sierminska et al., (2010, 2018), and Kapelle & Lersch (2020). Sierminska et al. (2018) report a mean gender gap in net worth (without pension wealth) across all households for Germany in 2012 of 40.2% if one takes men’s mean wealth as reference. In France Bonnet et al. (2014) found a mean gender wealth gap in 2010 of 12%, taking women’s wealth as reference and using the ‘Patrimoine’ dataset which has individual information but does not include pension wealth. For Estonia Meriküll et al. (2021) report a raw average wealth gap of 45%, with women’s value as reference, and including private pension wealth in the wealth measure. It needs to be highlighted that the gender taken as reference matters for the relative gap calculation, and this differs across studies. When mean wealth for men is greater, taking that as baseline produces a lower relative gap than using the women’s mean (or median) as reference. Meriküll et al. (2021), for example, find that men have 45% more wealth than women on average (baseline is women) in Estonia. This implies a 30% gap if men’s wealth is taken as the baseline (women have 30% less wealth than men).

The evidence currently available suggests that the mean gender gap in wealth may reflect for the most part what is seen towards the upper end of the distribution and be related to specific asset types, notably business wealth (Meriküll et al., 2021). As for the contribution of pension wealth, the limited existing literature already points to a clear distinction between public or statutory pension plans and private pension plans. Including components from private pension wealth has been found to generally increase the gender wealth gap. Rõõm & Soosaar (2020) find a large gender gap in assets held in in defined contribution plans for European countries participating in the HFCS survey (65% on average), a much greater gap than found in non-pension wealth with the same sources. Cordova et al. (2022), on the other hand, using data from the 2012 SOEP panel for Germany, find that considering pension wealth decreases the gender wealth gap from 35% to 28% of men’s average wealth, and that the effect is mainly due to statutory public pension schemes affecting the mid and lower part of the distribution. Statutory pension is also behind the practically non-existent gap in pension wealth entitlements found in Switzerland (Kuhn, 2020).

As far as drivers of gender wealth gaps are concerned, a range of factors has been suggested. Lifetime patterns of overall wealth accumulation appear to be flatter for women in Europe (Nutz & Gritti, 2022). Men may be more likely to hold sole ownership of assets in couples (Nutz, 2022), while differences in saving and investment behaviour between men and women are reflected in differences in portfolio composition and levels of returns (Neelakantan & Chang, 2010; Lersch, 2017), with men more likely to own business assets (e.g. Austen et al., 2014) and to hold riskier assets than women (Sunden & Surette, 1998). A contributory factor may be that women and men differ in risk attitudes, with women being more risk-averse (Bajtelsmit & Bernasek, 1996; Jianakopolos & Bernasek, 1998). Differences in the extent and nature of intergenerational wealth transfers may also play a role. Some studies find no significant differences in the amounts received by men and women (Ruel & Hauser, 2013) while others (Bartels et al., 2025) do find larger amounts are received by men, who also tend to receive them earlier. The literature also finds that sons are more likely to receive professional (business) assets and real estate while daughters are more likely to receive money (Bessière & Gollac, 2020). These assets are certainly more likely to contribute further wealth accumulation, also tending to be more protected from inheritance taxation (Tisch & Schechtl, 2023).

Still, the most important factor in explaining male-female differences in wealth accumulation appears to be their different labour market experiences, which become even more important when discussing pension wealth accumulation. Women’s lower labour market participation rate, their lower working hours, and the gender gap in hourly earnings all impair their overall wealth accumulation (Warren et al., 2001; Gardiner et al., 2016; OECD, 2023) and is also the dominant factor limiting their pension entitlements compared with men (Rõõm & Soosaar, 2020). The way women tend to cluster in particular occupational classes may also serve to hamper wealth accumulation, as investigated by Warren (2006) in the UK and by Waitkus & Minkus (2021) using SOEP wealth module data, and again be of specific relevance to pension wealth. The broader evolution of the extent and nature of women’s labour force participation has been set out in Goldin (1990), bringing out how this is intimately related to the role of women in caregiving and to the allocation of roles and tasks within families. A recent OECD study (OECD, 2025) finds that gender differences in employment, hours worked and hourly wages make a similar contribution to the gender gap in lifetime earnings (about one-third each), which averages 35% across OECD countries. The gender pension gap is associated with gender inequalities experienced at previous life stages and reflects cumulative gender inequality over the life course (Hammerschmid and Rowold 2019). While women’s shorter (full-time) employment duration has often been identified as the main driver of gender pension gaps (see for example Cordova et al., 2022, timing also matters: the recent study of Netherlands and Germany by Rowold (2025) finds that the duration, timing, and order of women’s care work experiences are more crucial predictors of pension entitlements. It has also been suggested that women may take less advantageous pension management choices, which could reflect financial literacy or different attitudes towards risk (Olivera & Iparraguirre, 2022).

### Pension wealth in Great Britain

The relatively disadvantaged situation of British women with respect to pension accumulation has been documented in Foster and Smetherham, (2013) and Cribb et al. (2023), who consistently find lower participation and contribution rates for women, especially in the private sector. This translates in lower estimated value of pension wealth (Gardiner et al., 2016; ONS, 2023).

As we have seen, the literature has generally found the gender pension gap to be more salient for private pensions. The weight of the public pension system is specially limited in Britain, with a replacement rate of 21.7% for the average worker (OECD, 2023, Table 4.2), half the OECD average and one of the lowest in Europe. The importance of the private pension system means that the Wealth and Assets Survey puts substantial effort into gathering in-depth information on private pension information, as described shortly, which the ONS then employs to produce ‘official’ estimates of pension wealth, whereas this is not done for public pensions.

It is important then to briefly describe the structure that underpins the particularly pronounced role of private pension provision in the UK. The statutory state pension system consists of a basic state pension and an earnings-related additional pension financed through earnings-related National Insurance contributions (NICs), together with means-tested Pension Credit. Comparative analysis shows that the UK state pension provides a lower level of income and income replacement than corresponding schemes in most other advanced (OECD) economies (see for example Harker, 2022; OECD, 2021a, 2021b). Operating alongside the state pension (the ‘first pillar’ in terms of the structuring in which pension systems are generally analysed), occupational (‘second pillar’) and personal (‘third pillar’) pensions play a substantially larger role in the UK than in most other European or OECD countries. This has been prompted and supported by a range of tax reliefs, both on contributions “on the way in” and on lump sum drawdowns “on the way out”. These have varied in structure and generosity over the years but play a major role in incentivising the provision of occupational pensions by employers and advantaging participating employees.

There are two main types of occupational schemes established by employers, defined-benefit (DB) pensions based on years of service and final pay or on career average revalued earnings and defined-contribution (DC) pensions based on contributions and the investment returns they generate. Employers now have a duty to enrol workers into such a pension, but the sector and size of the employer have long been a key determinant of whether an occupational scheme is provided and its nature. Employment in the public sector most often brings with it entitlement to an occupational pension with the amount to be paid related to salary. In the private sector large firms in the past often also operated DB, but recent years have seen a major move away from DB to DC schemes, reflecting the desire (and ability) of firms to transfer the risk associated with uncertain future pension costs to employees. Many large firms have wound down their DB schemes and transferred their workers to DC schemes over time, and smaller firms have either provided access to DC schemes or not offered and pension provision. Labour market trends such as outsourcing and growth in non-standard and marginal employment, have served to narrow the coverage of the occupational schemes of even large firms over time. Separately, personal pensions offer a private means of saving for retirement to those without access to an occupational scheme, with the government again providing tax relief (up to a limit) on contributions to such pensions. However, those on low wages and in non-standard and marginal employment are least well-placed to take advantage of these incentives and often lack coverage by occupational schemes, being thus most likely to have to rely entirely on the state for pension income in retirement. While distributed unequally as we shall investigate below, the overall importance of private pensions in the UK case is underlined by the fact that their value, as estimated by the ONS, now accounts for over two-fifths of total wealth when the wealth concept is extended to include them (see ONS, 2019b).

## Data and measures

The data we employ here is particularly rich in coming from a dedicated wealth survey gathering detailed data by asset type at the (mostly) individual adult level for a large representative sample repeated regularly over a significant and eventful period. Exploiting it represents a valuable addition to the literature as well as a case-study of particular interest. The Wealth and Assets Survey (WAS) is the official dedicated British household survey on wealth implemented by the Office for National Statistics (ONS) from 2006–2008. It has allowed the distribution of wealth in Great Britain to be compared with other countries (see for example Cowell et al., 2017, Balestra & Tonkin, 2018; Nolan et al., 2022) and anonymised microdata to be included in the Luxembourg Wealth Study (LWS) database The first wave carried out from mid-2006 to mid-2008, and follow-up waves have been released every two years since then. Our study then uses Waves 1 (carried out in 2006–08) to Round 7, which was carried out in 2018–2020^2^. We focus on the latest period (Round 7, 2018–2020) for our descriptive analysis in Section 4.1 and use all of the waves when we study the evolution of the gender wealth gap in Section 4.2, focusing on the first and last wave when analysing the change in the composition of the gap. We also compare the first and last wave from our data to highlight the changes in the composition of the gender gap specifically within pension wealth in Section 5. As for the decomposition analysis in Section 6, we focus on comparing the endowments and returns that explain the gap in Round 7 (2018–2020) with those of Wave 2 (2008–2010) due to many important labour market questions not being asked in Wave 1 (2006–2008).^3^

To increase the likelihood of including households towards the top of the wealth distribution an oversampling strategy based on geographical areas was employed by ONS.^4^ Even though the survey has a longitudinal component, the significant extent of attrition means that only a small proportion of the initial sample were successfully followed throughout and the sample had to be supplemented in successive waves so we concentrate here on exploiting the cross-sectional samples whose representativeness has been prioritised and assessed. For sample weighting purposes both cross-sectional and longitudinal weights are provided by the ONS at the personal (individual adult) level, and we thus use the cross-sectional weights for each wave to provide population-representative results.

The survey seeks in-depth information from respondents about wealth held in various forms and debts, as well as income and a range of demographic and other characteristics. The wealth measure covers real assets such as the main residence, other real estate property, vehicles, valuables such as jewellery, antique or art, and business wealth, as well as financial assets such as bank and other deposits, stocks and shares, voluntary private pension assets and whole life insurance policies. Net wealth comprises the aggregate value of all these assets held minus debt outstanding, in the form of mortgages, overdrafts, credit card debt, car loans, consumer loans, instalment and other loans. Although the value of public pension wealth rights is not included, the individual value of occupational (employer managed) and personal pension wealth is also provided in WAS. This pension wealth component is distinguished and included in the ‘official’ wealth distribution estimates produced from WAS by the ONS (see for example ONS, 2019b). We explain below the details of the pension wealth measure and the rich disaggregation by type of plan that we use in this study.

Some of wealth information is obtained at the household level in WAS: the main residence and its contents, valuables (art, jewels, etc.) and vehicles. To obtain individual wealth aggregates we here split the value of the main residence, its contents, valuables and vehicles equally among the two members of each couple in non-single households.^5^

The responses of all other elements of wealth are at the individual level, the relevant questions being part of the individual questionnaire to which each adult in the household was asked to respond. These are as follows:

- Net *financial wealth*: bank accounts, stocks, bonds and investments, loans to others, net of all financial debt (loans owed, credit card debt…);
- Net self-employed *business wealth* (market value of business);
- Net *‘other property’ wealth* (real estate other than main residence and its contents);
- *Pension wealth* (occupational and private individual pension plans).

Aggregating all components, we are able to obtain a measure of individual wealth for each individual in WAS over 30 years of age. We do not use younger population to allow some labour market experience which is necessary to accrue relevant pension wealth value. Negative wealth values are relatively scarce, representing 0.55% of the observations for women, and 0.42% for men in the 2018–2020 dataset. These values are kept in the sample, as we do not perform any analysis incompatible with negative values. In the decomposition exercise in Section 6, values are Inverse Hyperbolic Sine transformed, which reduces the influence of extreme values in the estimates while allowing to keep negative observations.

In WAS an extensive set of questions is employed to capture the information required for private pension wealth to be estimated, all at the individual level. The information sought covers whether the respondent is currently a member of an employer’s occupational pension scheme, if so for how many years, details on that scheme including the name of the scheme, the contributions being made by the employee and employer, and whether the pension amount due is based on contributions to the scheme and the rate of return achieved on their investment or is salary-related. If the pension is based on contributions and the return on them (i.e. it is a DC scheme), the respondent’s estimate of the current value of their pension pot is sought. If the scheme is salary-related (i.e. it is a DB scheme), details are sought on the basis on which that is determined in terms of proportion of final or averaged salary and if the latter the period over which it is averaged. Similar information is sought on any other employer/occupational pension schemes to which the respondent and/or an employer is contributing or has contributed in the past. Separately, details on any personal or stakeholder pension schemes to which the respondent is contributing or has contributed in the past are also sought, including the name of the scheme, the name of the provider, the scale of contributions being made or previously made by respondent and by an employer (if any), and the current value of the pension pot. Across those schemes details of any future lump sum entitlements or lump sums previously withdrawn as well as any annuities purchased are asked. Note that due to the detail and precision required to report pension contributions, we have only kept in the sample in which the individual reported the data in-person and not by proxy.

While there is evidence in the literature that the general level of financial literacy may be lower on average among women than men (something also found in our sample; see Table 5 in Section 6) this may not translate into reporting bias. Some studies find lower general financial literacy does not extend to knowledge about own individual finances.^6^

The estimate of private pension wealth constructed by ONS based on this information, on which we rely here in our analysis, relates to value of any/all private pension pots already accrued, distinguishing occupational defined benefit, occupational defined contribution, and personal pensions. As well as schemes to which the individual is currently contributing, this includes both retained rights in previous pensions and pensions already in payment.^7^ The estimated current values for occupational DC pension pots are primarily based on the values provided directly by respondents. For occupational DB pensions, the current value is estimated as the equivalent DC pension pot that would be needed to buy (at the date the individual responded to the survey) the regular income expected from the pension on retirement based on the discount and annuity rates prevailing at the interview date. Finally, personal pensions purchased from an insurance company by an individual and self-invested personal pensions are valued in the same way as occupational DC pensions. In all cases the estimates include only the pension rights accumulated to date; for people who are still working, they do not include rights which may be built up between then and when the person retires. The estimates relate solely to private pensions, not the state retirement pension (basic or earning related) to which entitlement is earned via social insurance contributions.

## The gender wealth gap in Great Britain in 2018–20 and the role of pension wealth

We now employ the WAS data described in the previous section to provide an up-to-date picture of the extent and nature of the gender wealth gap in Great Britain with and without private pension wealth in the latest wave of our sample (Round 7) in which households were surveyed in 2018–2020. We see on Table 1a that when private pensions are included men, on average, have mean wealth of approximately £469k while women have approximately £310k.

**Table 1:** Wealth Levels and Gender Gaps, Great Britain, 2018–20

*1a. Wealth levels and wealth gap by gender and couple status including pension wealth*										
		Wealth Levels					Wealth Gaps: Absolute (£ x 1000) and Relative (% of men’s wealth)			
		Mean (£ x 1000)		Median (£ x 1000)			Mean		Median	
		Estimate	S.E.	Estimate	S.E.		Estimate	S.E.	Estimate	S.E.
All	Men	469	10	246	4	Gap	159	7	67	6
	Women	310	3	180	5		34.0%	***	27.1%	***
Couple	Men	502	12	273	7	Gap	201	13	87	9
	Women	302	5	186	4		39.9%	***	31.9%	***
Single	Men	383	12	187	8	Gap	59	14	25	11
	Women	324	8	162	8		15.4%	***	13.5%	**

*1b. Wealth levels and wealth gap by gender and couple status excluding pension wealth*										
		Wealth Levels					Wealth Gaps: Absolute (£ x 1000) and Relative (% of men’s wealth)			
		Mean (£ x 1000)		Median (£ x 1000)			Mean		Median	
		Estimate	S.E.	Estimate	S.E.		Estimate	S.E.	Estimate	S.E.
All	Men	241	6	136	2	Gap	39	7	12	3
	Women	202	3	123	2		16.3%	***	9.2%	***
Couple	Men	249	9	141	2	Gap	51	9	11	4
	Women	198	4	130	2		20.4%	***	7.6%	***
Single	Men	223	7	104	8	Gap	14	9	8	10
	Women	209	5	96	7		6.1%	*	7.3%	

Individual total net wealth and individual total net wealth excluding pension wealth for the population of 30 years of age and older. Singlehood refers to single, separated, divorced and widowed individuals not currently living with a partner. Data from Round 7 of Wealth and Assets Survey (collected 2018–2020). Standard Errors calculated via bootstrap (standard deviation of bootstrapped samples). Significance of the gap based on the distribution of the bootstrap estimates and using the t-statistic, *** indicates statistical significance at the 1% level, ** at the 5% level, and * at the 10% level

The gender gap between these means amounts to 34.0% of men’s average wealth. Compared with previous studies reviewed in Section 2, this gap for Great Britain is a good deal higher than the average for the twenty-two EU countries analysed by Kukk et al. (2023) using HFCS data, which was 24%; six of these countries were estimated to have mean gaps above 30% (namely Finland, France, Greece, Ireland, Luxembourg and Malta. It should be emphasised however that their figures are based on estimates of individual wealth from household-level aggregates and individual characteristics rather than the individual-level reports we have been able to use for Great Britain; the age range included is also not identical so the comparison should be taken as indicative. Our estimate for Great Britain is similar to the 30% gap (when measured using men’s wealth as baseline) found for Estonia by Meriküll et al. (2021) using also individual wealth data.

As mean wealth levels and thus gaps will be heavily influenced by the small number of cases with very high wealth, it is important to complement them with the corresponding median-based figures which present a more representative picture. The median gap is smaller in magnitude and in relative terms for average than for mean values, although still relevant. When considering median wealth, the overall gender gap is 27.1% of men’s median wealth. This difference between median and mean-based gaps points to the importance of patterns towards the top of the distribution, where large absolute gaps influence the mean substantially as we shall see below; the median is more representative of the situation of a ‘typical’ person, but both mean and median are of course valuable indicators.

It should be noted that widows and widowers not living with a partner are included among ‘singles’ in this analysis, but are distinctive in presumably having received the full assets of their spouse on the latter’s death; the fact that there are more widows than widowers means women will have gained disproportionately, serving to reduce the gender wealth gap. We have investigated the role this plays by excluding widowed individuals from the sample for analysis, and results presented in Appendix B show that the mean wealth gender gap for the overall excluding pensions is then one-fifth higher while the gap including pensions is about one-tenth higher.

To bring out the role of private pension wealth in these gender gaps the Table 1b shows the corresponding figures for wealth measured as net worth, i.e., not including pension wealth. We see first that mean and median wealth levels are then much lower for men – their mean wealth falls from about £469k to £241., so private pension wealth constitutes almost half of the ‘wealth including private pensions’ aggregate for men. We then see that excluding private pension wealth brings down mean wealth for women from £310k to £202k, private pension wealth making up one-third of total wealth including private pensions for women. As a consequence, the gender wealth gap falls substantially when private pensions are excluded, with the gap between mean wealth for women versus men down to 16.3%, and the gap between median wealth for women versus men then 9.2%. Failure to incorporate private pension wealth thus understates the gender gap for Great Britain very substantially.

### The gender wealth gap by age and couple status

Distinguishing individuals on the basis of their marital/partner status, Table 1 also reveals that - for the whole sample of those over 30 years old - the gender wealth gap is more pronounced among men and women living in couples than among single individuals. Including private pensions in wealth, the gap between mean wealth for women versus men in couples is close to 39.9% whereas among single individuals it is much more modest at 15.4%; that pattern is similar when looking at median rather than mean wealth levels (31.9% and 13.5% respectively). When private pensions are not included the gap between means is again more pronounced among individuals in couples, but the opposite is the case for the gap based on median wealth, suggesting that patterns towards the top – which will have a pronounced impact on the mean but not the median – may be distinctive.

Previous studies have also found the gender wealth gap to be predominantly in couple rather than single-adult households. the comparative study by Kukk et al. (2023) concluded that the gap in multi-member households drives the overall gap observed, which is also the conclusion of Meriküll et al.’s (2021) study for Estonia. A number of factors have been advanced to explain this pattern for net wealth other than pensions, with differences between male and female partners in business and financial assets playing a central role and those gaps being pronounced at the upper end of the net wealth distribution. As Schneebaum et al. (2018) bring out, different selection of men and women into marriage/cohabitation also has to be taken into account in understanding this pattern. Marriage is associated with greater wealth but is also endogenous with respect to wealth; being married leads to faster accumulation of wealth independently of other characteristics (Ruel & Hauser 2013), but at the same time wealthier individuals or people who have better potential for wealth accumulation are more likely to marry. In terms of the ‘marriage premium’, women tend to see a lower premium in financial assets in particular than men (Lersch 2017). As far as pension wealth is concerned, differences in labour market experiences were noted in our review of the literature above as key, and differences in that respect between partners – underpinned by the impact of having children and the uneven distribution of caregiving more generally – produce the marked differences observed in pension wealth. It has also to be emphasised that single adult households are a quite heterogenous group, including single people who have never married and those who are widowed or divorced as noted above.^8^

Age is a key factor in wealth accumulation, so it is of particular interest to examine in Table 2 how wealth levels and gender wealth gaps vary among couples and single individuals distinguishing three different age groups, again for net wealth both including (Table 2a) and excluding (Table 2b) pension wealth. When pension wealth is included both wealth levels and the gender gap are seen to increase with age among couples, whether the mean or the median is used. Among single individuals mean wealth levels also go up with age for both men and women, as does the absolute gap between means when pension is included, although the relative gap (in %) is highest for the youngest of the age groups distinguished. When pensions are not included, gender gaps between the means in absolute and relative terms are actually highest for the younger age range among both couples and single individuals, narrowing as one moves up the age range.

**Table 2:** Wealth Levels and Gender Gaps, Great Britain, 2018–20

*2a. Wealth levels and wealth gap by gender, age and couple status including pension wealth*										
		Wealth Levels					Wealth Gaps: Absolute (£ x 1000) and Relative (% of men’s wealth)			
		Mean (£ x 1000)		Median (£ x 1000)			Mean		Median	
		Estimate	S.E.	Estimate	S.E.		Estimate	S.E.	Estimate	S.E.
***Couples***										
30–44	Men	214	19	112	6	Gap	66	21	17	7
	Women	147	5	95	3		31.1%	***	14.8%	***
45–59	Men	540	19	314	12	Gap	176	22	79	15
	Women	364	10	235	8		32.6%	***	25.1%	***
60+	Men	685	20	427	10	Gap	281	22	148	12
	Women	404	9	279	7		41.0%	***	34.6%	***
***Singles***										
30–44	Men	162	12	100	14	Gap	48	14	67	15
	Women	114	8	33	3		29.6%	***	67.3%	***
45–59	Men	378	27	175	15	Gap	65	33	47	21
	Women	313	15	128	15		17.1%	**	27.1%	***
60+	Men	513	18	307	16	Gap	105	21	30	18
	Women	409	11	277	10		20.4%	***	9.9%	*

*2b. Wealth levels and wealth gap by gender, age and couple status excluding pension wealth*										
		Wealth Levels					Wealth Gaps: Absolute (£ x 1000) and Relative (% of men’s wealth)			
		Mean (£ x 1000)		Median (£ x 1000)			Mean		Median	
		Estimate	S.E.	Estimate	S.E.		Estimate	S.E.	Estimate	S.E.
***Couples***										
30–44	Men	142	18	67	4	Gap	38	19	0	4
	Women	104	4	67	2		26.5%	***	0.2%	
45–59	Men	249	15	141	4	Gap	34	17	−5	6
	Women	215	7	146	4		13.6%	**	−3.9%	*
60+	Men	327	12	214	4	Gap	46	15	11	6
	Women	281	7	203	4		14.2%	***	5.1%	*
***Singles***										
30–44	Men	121	9	64	13	Gap	39	12	40	12
	Women	82	7	24	2		32.3%	***	62.5%	***
45–59	Men	188	12	70	15	Gap	23	15	12	18
	Women	165	9	58	9		12.1%	*	17.3%	
60+	Men	307	12	185	11	Gap	25	14	−15	14
	Women	282	8	201	7		8.1%	*	−8.3%	

Estimation of individual total net wealth and individual total net wealth excluding pension wealth for the population of 30 years of age and older, split by the indicated age-groups. Singlehood refers to single, separated, divorced and widowed individuals not currently living with a partner. Data from Round 7 of Wealth and Assets Survey (collected 2018–2020). Standard Errors calculated via bootstrap (standard deviation of bootstrapped samples). Significance of the gap based on the distribution of the bootstrap estimates and using the t-statistic, *** indicates statistical significance at the 1% level, ** at the 5% level, and * at the 10% level

### The mean gender wealth gap by components and over time

We now use the WAS data to examine how the average wealth gap in Great Britain evolved over the period from 2006–08 to 2018–20. In Table 3 we present the values of the absolute and relative gaps for total wealth and for each component in the first and last waves analysed. Substantial gaps are observed in total wealth, pension wealth, and financial and business wealth. In 2020, the absolute gap in total wealth stood at £159.3k, representing a considerable increase from £105.7k in 2008; relatively, this gap widened from 27.3% to 34.0% over the period, women held significantly less wealth than men and this disadvantage grew. Pension wealth displayed the largest relative gap in 2008 (57.0%) which slightly narrowed to 52.7% by 2020, although the absolute gap increased substantially from £86.9k to £119.9k. Financial and business wealth also show a major disparity, with the absolute gap doubling from £18.3k to £36.7k and the relative gap surging from 25.1% to 47.3%.

**Table 3:** Mean gender wealth Gap in total wealth and in each main wealth component, WAS 2006–08 and 2018–20

		2008	2020
Total Wealth	Mean Men	387.1	468.9
	Mean Women	281.4	309.7
	Absolute Gap	105.7	159.3
	Relative Gap	27.3%	34.0%
Financial and Business Wealth	Mean Men	72.7	77.6
	Mean Women	54.5	40.9
	Absolute Gap	18.3	36.7
	Relative Gap	25.1%	47.3%
Main Residence Wealth	Mean Men	108.5	119.6
	Mean Women	113.8	118.9
	Absolute Gap	−5.2	0.7
	Relative Gap	−4.8%	0.5%
Pension Wealth	Mean Men	152.4	227.5
	Mean Women	65.5	107.6
	Absolute Gap	86.9	119.9
	Relative Gap	57.0%	52.7%
Physical and Other Property wealth	Mean Men	53.4	44.3
	Mean Women	47.6	42.3
	Absolute Gap	5.8	2.0
	Relative Gap	10.9%	4.4%

Estimation of individual total net wealth for the population of 30 years of age and older. Data from Wave 1 and Round 7 of Wealth and Assets Survey (collected 2006–2008 and 2018–2020 respectively)

In contrast, gender differences in main residence wealth and physical/other property wealth were less pronounced and showed different dynamics. The gap in main residence wealth was minimal in both waves, even slightly favouring women in 2008 (absolute gap −£5.2k, relative gap −4.8%) before shifting to a negligible disadvantage in 2020 (£0.7k, 0.5%). Physical and other property wealth saw a reduction in the gender gap over the period, with the absolute difference decreasing from £5.8k to £2.0k and the relative gap narrowing from 10.9% to 4.4%. Overall, while minor convergence occurred in property-related wealth, the widening gaps in total, financial, and pension wealth underscore enduring structural disadvantages faced by women in accumulating significant forms of wealth between 2008 and 2020.

**Fig. 1:**
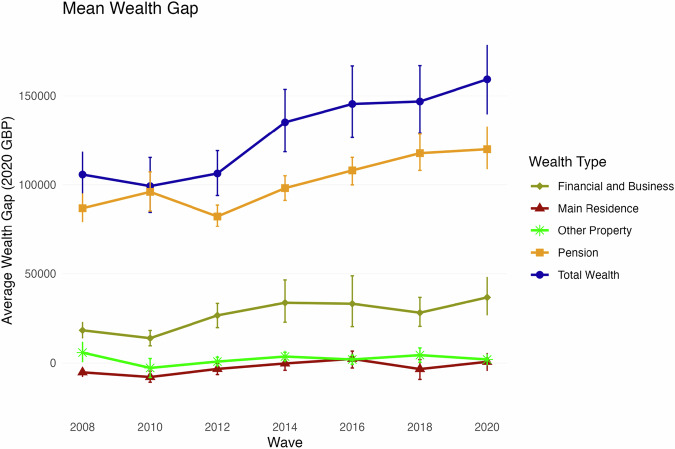
**Gender wealth gap in total wealth (including pension wealth) and in each main wealth component, WAS 2006–08 to 2018–20**. Estimation of individual average net wealth gender gap for each wealth component for the population of 30 years of age and older in Waves/Rounds 1–7 of the Wealth and Assets Survey. Waves are marked in the X-axis by the latest year in which data was collected. Standard errors obtained by bootstrap (n = 500). Other Property includes all property besides the main residence and physical wealth (valuables, household contents, cars, etc). Values in absolute real GBP (2020 prices)

Figure 1 shows the evolution of the absolute gap for total wealth and for its different components. Note wealth gaps are expressed in real terms, adjusted to 2020 prices throughout. The gap for private pension wealth (blue line with the square marker) stands out as the most pronounced. This gap has increased steadily since 2012, and throughout the period represents the most substantial component of the difference in average wealth between men and women.

The financial and business wealth gap is less but still substantial, with men’s mean wealth exceeding that of women by approximately £20k to £40k. This gap has fluctuated slightly but overall also trended upward. The story changes when we consider the main residence category where the wealth gap is slightly negative, with women’s mean wealth exceeding that of men by about £5k in the first period considered, although it shows a decreasing trend. The ‘other property’ component of wealth shows a modest advantage for men with little variation over time.

**Fig. 2:**
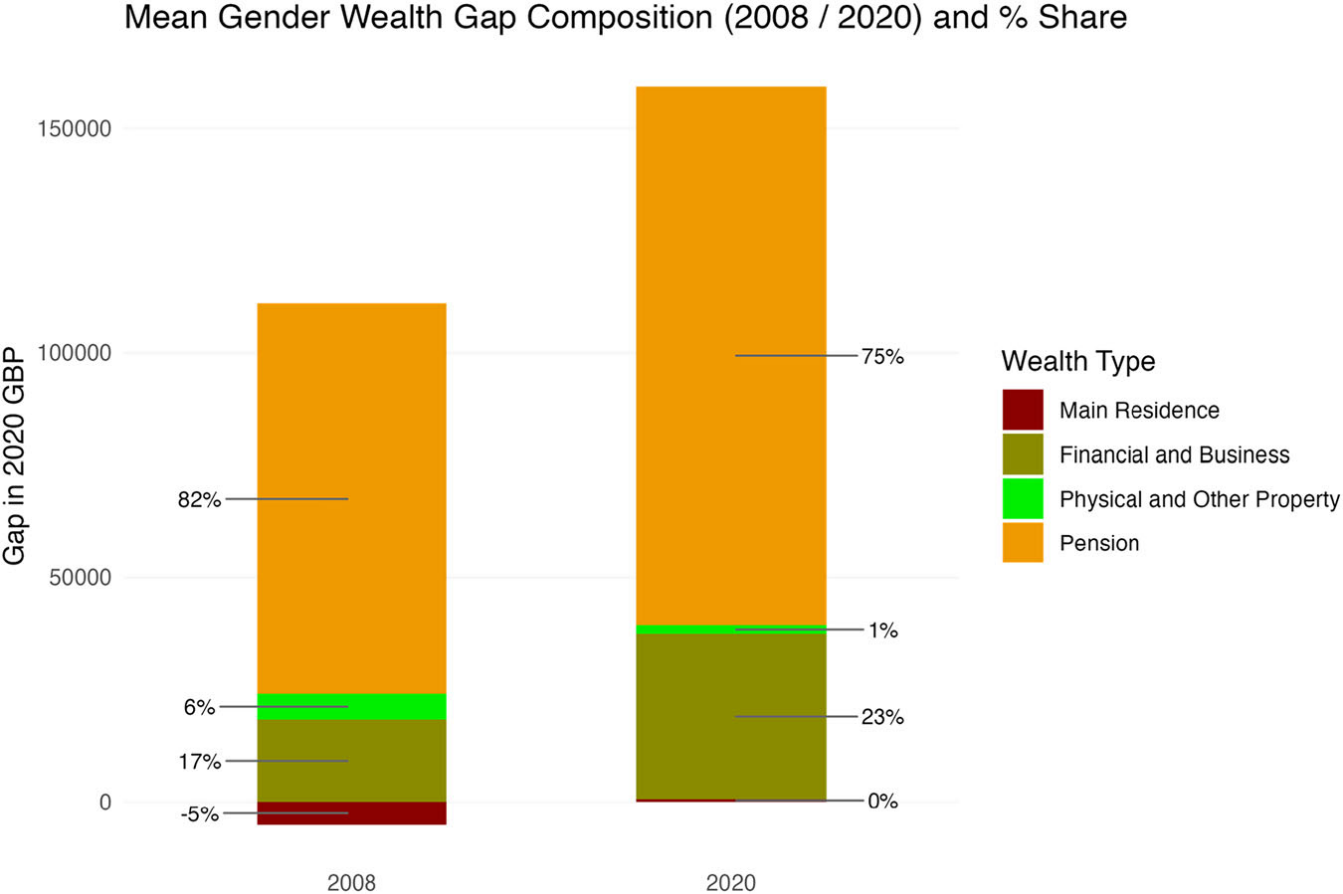
**Contribution of each wealth component to the Gender gap in total wealth in the first and latest available waves (2006–08 and 2018–20)**. Average gender wealth gap (aggregate total and coloured by components) for all population of 30 years of age and older for Wave 1 (2006–2008) and Round 7 (2018–2020) of the Wealth and Assets Survey. Values in absolute real GBP (2020 prices) and share of total absolute gap of each component expressed in percentage

Overall, the aggregation of these components into total wealth shows that the overall gender wealth gap has increased substantially over the period (in real terms), largely driven by the private pension and financial/business wealth components. Figure 2 brings this out by comparing the composition of the gender wealth gap by wealth type at the start and end of the period. Between 2006–08 and 2018–20 the gender gap in pension wealth increased by around £33k and that for financial and business wealth increased by £18k, with those two components accounting for almost all of the £54k increase in the overall wealth gap over the period. For main residence wealth the modest gap in favour of women narrowed and its virtually zero in the last period considered, offset by a similar narrowing in the gap in favour of men for other property wealth. Private pension wealth clearly plays the dominant role in the gender wealth gap throughout, followed by financial and business wealth.

In both the 2006–08 and 2018–20 periods, the gap in pension wealth accounted for most of the total average wealth gap between men and women, contributing 82% initially and remaining the primary driver at 75% in the later period, despite a substantial increase in the total absolute gap. The contribution of financial and business wealth to the overall disparity grew markedly, rising from 17% to 23%, becoming an increasingly significant factor in gender wealth inequality. In contrast, differences in property wealth played a much smaller role; the main residence wealth gap was negligible or even slightly favoured women initially (reducing the total gap), while the contribution from other property wealth diminished to just 1% by 2018–20. Overall, the widening total wealth gap observed over the decade appears largely attributable to the substantial and growing absolute differences in pension and financial assets.

### The gender wealth gap in great britain across the wealth distribution

**Fig. 3:**
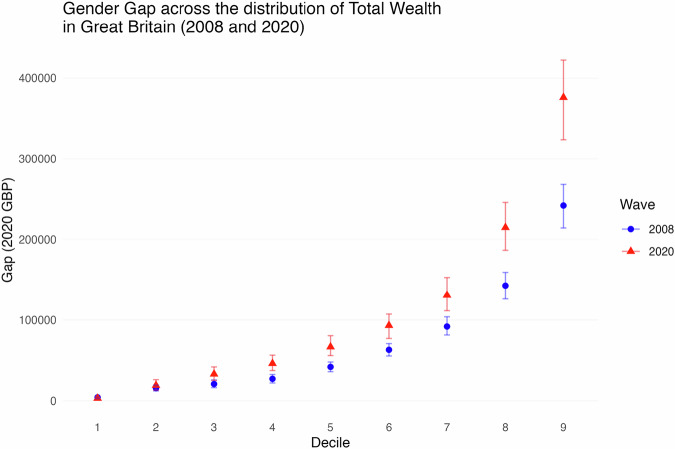
**Gender Wealth Gap at different deciles of the total wealth distribution in Great Britain in 2006–08 and 2018–20**. Gender Wealth Gap at different deciles of the total wealth distribution in WAS Wave 1, 2006–2008 represented in blue dots, and in Round 7, 2018–2020), represented in red triangles. Error bars represent 95% confidence intervals obtained by bootstrap replication of the estimation at each decile

The fact that the mean gender wealth gap exceeds the median wealth gap as we have seen above already points to the marked influence of the gap at the upper tail of the wealth distribution. To clearly assess that effect, we now look at how the wealth gap in Great Britain varied across the wealth distribution over the period from 2006–08 to 2018–20. Figure 3 shows the overall wealth gap (including pensions) in absolute terms, expressed in £ values at 2020 prices, at deciles of the wealth distribution for different components in the 2006–08 and 2018–20 surveys. Focusing first on the top of the distribution, the gap for those at the cut-off for the wealthiest 10% was already substantial in the first wave at almost £250k. By 2018–2020 this gap has expanded considerably to over £350k, with the statistical significance of that increase attested by the 95% confidence intervals also shown.

Though much greater, the wealth gap is not however confined to the wealthiest, with noticeable gaps all across the distribution except at the first decile. Both the gap levels and the gap change across waves increase as we go up the distribution. Note these increases in the absolute wealth gap are all in 2020 prices, which reflects an increase of the gender wealth gap in real terms.

**Fig. 4:**
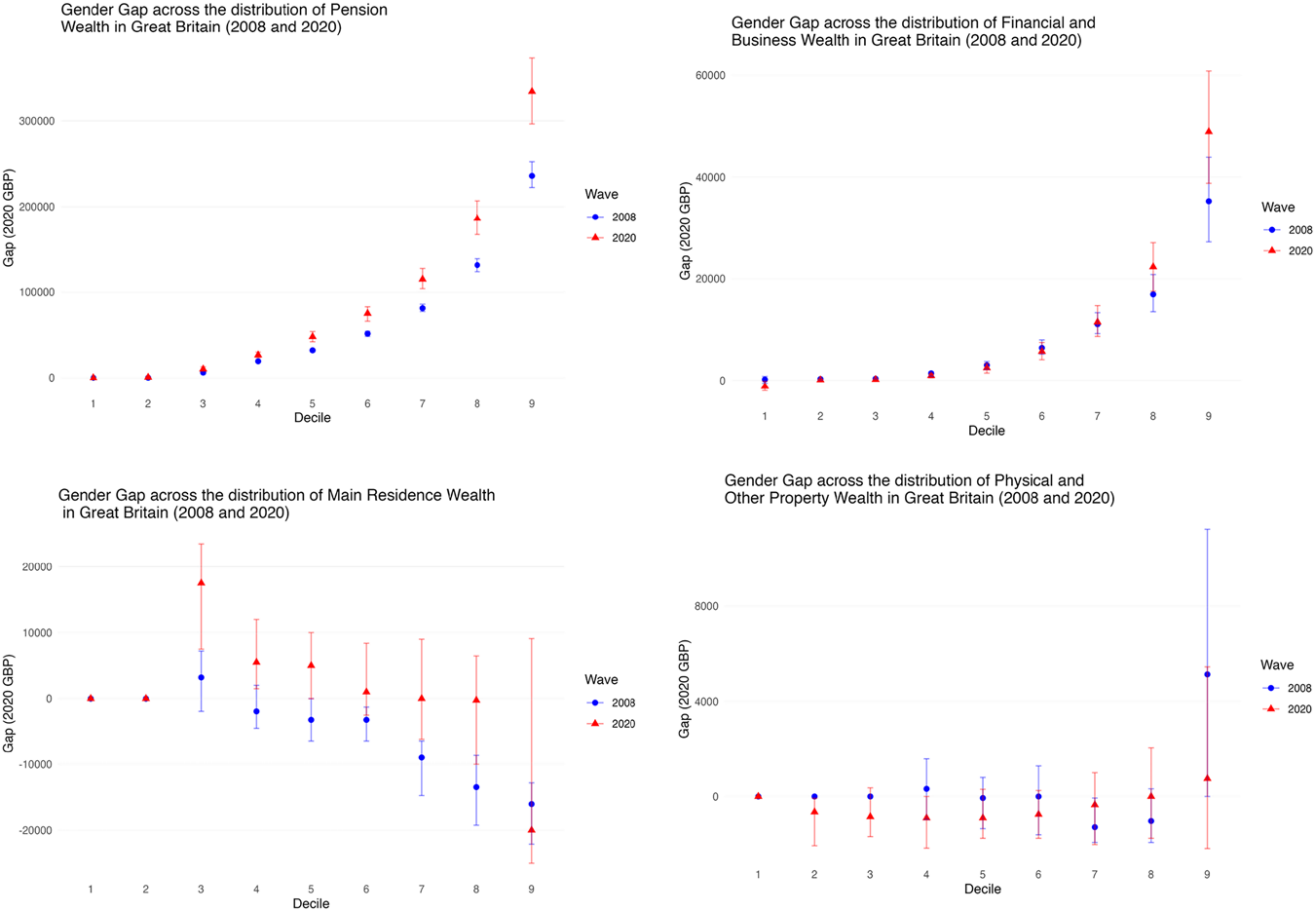
**Gender Wealth Gap at Different Deciles by Wealth Type, WAS 2006–08 and 2018–20**. *the first WAS wave (Wave 1, 2006–2008), represented in blue dots, and the latest available wave (Round 7, 2018–2020), represented in red triangles. Error bars represent 95% confidence intervals obtained by bootstrap replication of the estimation at each quantile*. Note the scale is different in each graph to help visualization

It is also informative to look separately at the gaps across the wealth distribution for each of the main asset types involved. Figure 4 shows that for pension wealth the gender gap (between means) at the top decile cut-off already exceeded £200k in 2006–2008, and by 2018–20 had increased by more than another £100k. At the median there was also a substantial gap, which again increased over the period. Financial and business wealth also shows a pronounced gender gap towards the top, amounting to about £50k in 2018–20 in the tenth decile. Main residence wealth is distinctive with some gap favouring women across the deciles but particularly towards the top in 2008, although narrowing over time. Other property displays no significant gap in the distribution.

The confidence intervals indicate varying degrees of statistical significance for these estimates with tighter intervals at the top suggesting more precise estimates of the gap, especially for pension wealth. The overall wealth gap increases as one moves up the wealth deciles, with pension wealth and financial and business wealth the most significant contributors to the gap towards the top.

## The pension wealth gender gap in the active population: the role of different types of pension plans

We have seen that the gap in private pension wealth represents a substantial proportion of the overall gender wealth gap in Britain and has been growing in recent years. We have emphasised from the outset that occupational and personal pensions – ‘pillars two and three’ - play a substantially larger role in the UK than in most other European or OECD countries. Furthermore, the role of DB versus DC schemes and how that has been evolving is central to what has been happening within ‘pillar two’. Not only the role of occupational pensions but the proportion of assets in DB versus DC schemes within them varies greatly across those countries, with Finland and Norway for example having traditional DB schemes, hybrid schemes are dominant in countries, such as Belgium, Germany, the Netherlands, Luxembourg, Portugal, Spain, and Sweden, whereas many central and eastern European countries operate only DC schemes in the second pillar and their share is also particularly high in countries such as Austria, Greece, France, Iceland, and Italy. A shift towards DC schemes at the expense of DB has been seen in various countries including Ireland, Denmark, and Sweden over the past 15–20 years, with new and younger employees usually covered by new DC schemes but older employees remaining covered by DB schemes (Pensions Europe, 2024). Indeed, the Dutch pension fund, by far the largest in the euro area, is in transition from DB to be fully DC-based, which will bring the share of DC pension fund schemes in the euro area from around 17% to 77% (Rousová et al., 2021). Factors identified as driving this shift include changes in accounting standards and solvency and funding rules, increasing life expectancy, structural changes in the labour market, and declining interest rates to recently making it difficult for DB schemes to generate sufficient returns to meet their obligations; companies are also choosing DC schemes as they offer greater control over pension costs and less exposure to the risks and volatility associated with DB schemes (Pensions Europe, 2024). The UK has seen a marked shift away from DB schemes, due to such factors as well as some more specific ones such as changes in tax treatment; in addition, the proportion of employees with workplace pensions has increased steadily since 2012 due to the introduction of automatic enrolment and this has for the most part been into DC schemes. DC pensions overtook DB pensions for the first time in 2019 in terms of number of employees covered, with the majority of DB schemes now closed to new members (Davies et al., 2025).

We now delve in more detail into the nature of the pension wealth gap in Great Britain and how it has been changing, focusing in particular on the role of different types of scheme. For this analysis we make use of the wealth valuations for different type of pensions calculated by the ONS with WAS data, which includes all types of pension wealth except public pension rights. This distinguishes occupational from personal pensions, and also the pension rights obtained from pensions already being received by the respondent (pensions in payment) and those from a deceased spouse. As outlined earlier the valuation of DC contribution plans is based on the current value of the pension pot reported by the respondent, while for DB plans and pensions already in payment the actuarial value of the plan is estimated based on the amount received or promised and the discount and annuity rates at the time of response.^9^

Our analysis above of the gender wealth gap across the distribution covered all over 30 year-olds. Now, when focusing on pension wealth it is informative to present estimates only for those aged 30–65 who are still active in the labour market/not in receipt of a private pension, to focus on future pension prospects. This excludes those already receiving ‘pension in payment’, a component for which we cannot know the type of plan that formed that wealth, and allows us to focus on the composition of private pension wealth. Among the 30–65 active group, we split the sample between 30–45 and 46–65-year-olds to compare those building their pension in their earlier years in the labour market and those in the later stages of the labour life cycle.

**Fig. 5:**
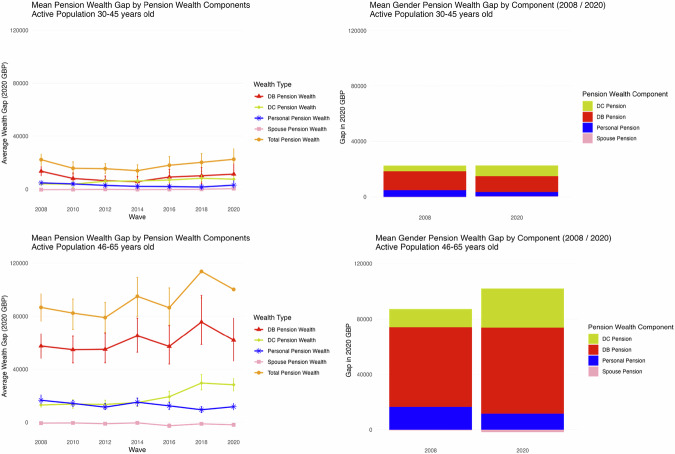
**Evolution and composition of pension wealth gap for active population (age groups 30–45 and 46–65)**. Evolution of mean pension wealth for total pension wealth and for each pension wealth component across the seven waves of WAS (2008–2020) in the left part of the graph. Top-left shows this evolution through all of the seven waves for the 30–45 age group, while bottom left shows the evolution for the 46–65 age group. The right-hand side bar-graphs show the share of each component in the total pension wealth gap in 2008 and 2020, for the 30–45 age group (top right) and for the 46–65 active age group (bottom right)

Figure 5 and Table 4 show that the gender gap in constant 2020 prices has remained stable between the first and last WAS wave (analysed here) for those at the earlier years of the work career, but the composition has changed, with the DC plans now accounting for a larger share of the gap. At later stages of the work cycle there is an increase in the gender pension wealth gap, driven mostly by increase in the gap in DC pension wealth. The gap occurs in all plans, but the increase over time is due mostly to the gaps in DC pension wealth.

**Table 4:** Mean gender pension wealth gap for total pension wealth and for each pension wealth component in Wave 1 (2006–08) and Round 7 (2018–20) in WAS

Active Pop. Age 30–45				
Pension Wealth Type	Gap (£)	Contribution to Total Pension Wealth Gap (%)	Gap (£)	Contribution to Total Pension Wealth Gap (%)
	2008		2020	
DB Pension Wealth	13597	61%	11408	51%
DC Pension Wealth	4105	18%	13907	62%
Personal Pension Wealth	4833	22%	3109	14%
Spouse Pension Wealth	−270	−1%	467	2%
Total Pension Wealth	22266	100%	22581	100%

Active Pop. Age 46–65				
Pension Wealth Type	Gap (£)	Contribution to Total Pension Wealth Gap (%)	Gap (£)	Contribution to Total Pension Wealth Gap (%)
	2008		2020	
DB Pension Wealth	57504	66%	61910	62%
DC Pension Wealth	13106	15%	28333	28%
Personal Pension Wealth	16669	19%	11806	12%
Spouse Pension Wealth	−533	−1%	−1787	−2%
Total Pension Wealth	86747	100%	100261	100%

Gender wealth gap for main pension wealth components in Wave 1 (2006–08) and Round 7 (2018–20) in WAS for all population and for the active (not-retired) population in the 30–45 and 46–65 age groups

While the widening absolute gap highlights the growing disparity in real monetary terms, it is also useful to consider the evolution in relative terms. In Appendix Fig. 9, we present the relative pension wealth gap for the active population. By age groups, the older group shows a slight decline in the relative pension wealth gap, but the opposite occurs for the younger group. This reveals that, overall, the relative disadvantage has remained rather stable over the period, suggesting that women’s pension wealth has grown at a similar rate to men’s, albeit from a much lower base (which is consistent with the widening of the absolute gap).

**Fig. 6:**
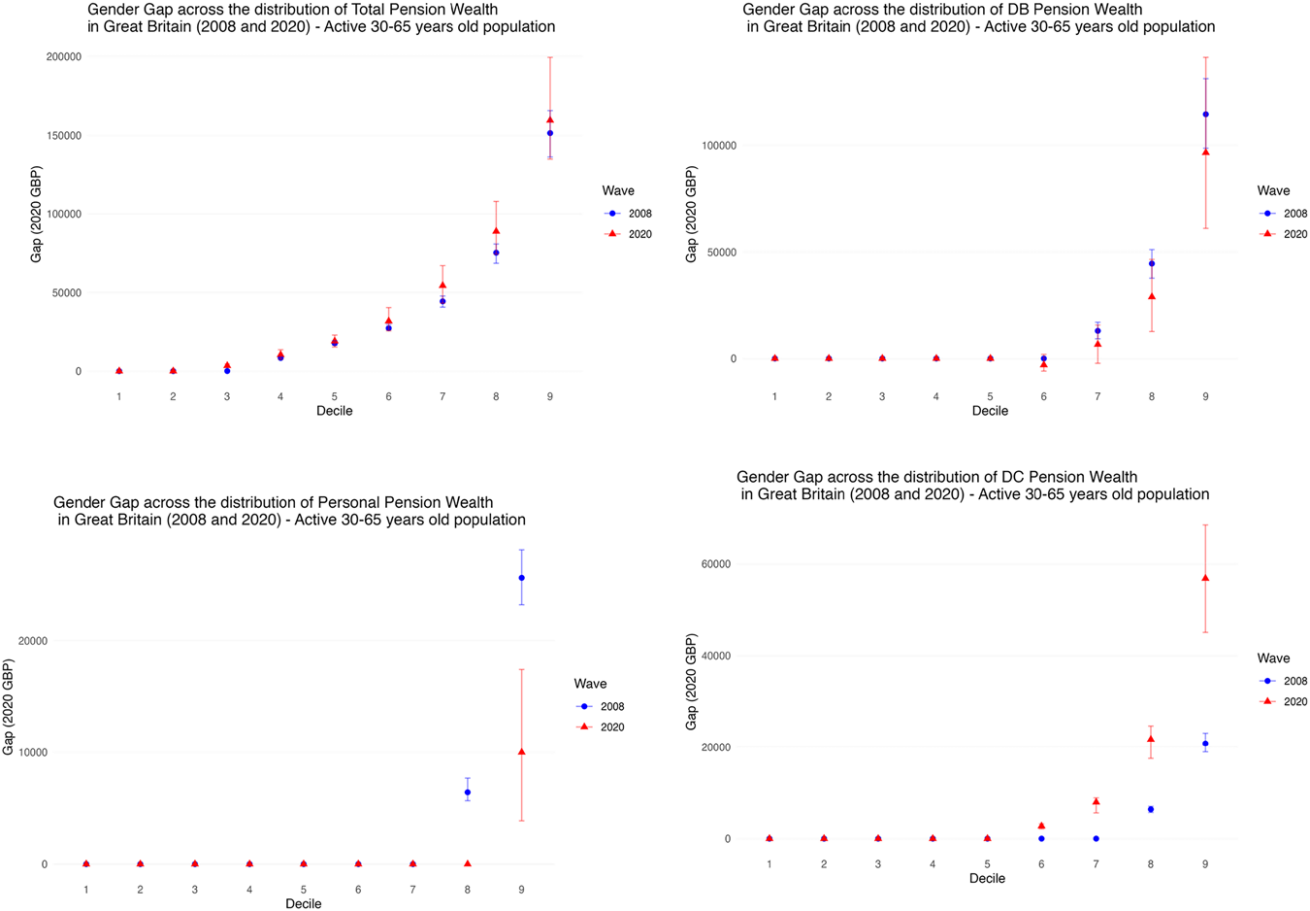
**Gender wealth gap at each decile of the distribution for total pension wealth and for main pension wealth components, WAS 2006–08 and 2018–20. Active Population 30–65 years old**. Gender wealth gap at each decile of the distribution for total pension wealth and for main pension wealth components in Wave 1 (2006–08) and Round 7 (2018–20) in WAS, for the active (not-retired) population in the 30–65 age group. Top-left shows the total pension wealth gap, top right the gap in DB pension wealth gap, bottom left the gap in DC pension wealth and bottom right the gap in Personal pension wealth. Note the scale is different in each graph to help visualization

Figure 6 shows how the private pension gap varies across the wealth distribution in 2006–08 and 2018–20 for the whole of the 30–65 not retired population.^10^ In total pension wealth and in all wealth components the main differences between men and women occur at the top of the distribution. For total pension wealth (top-left in Fig. 6) the gap is marginal towards the bottom and increases when moving up the distribution, reaching over £150k at the cut-off for the top 10%. That gap has slightly increased everywhere in the period analysed, but especially at the top of the distribution, where the increase in the gender gap for wealth held in DC schemes (bottom-right quadrant) has been particularly pronounced.

These results bring out the salience for the gender pension wealth gap in Great Britain of the major structural changes in the pension landscape that have been seen over recent decades, notably the declining importance of DB schemes and the corresponding enhanced role being played by DC schemes. They also highlight the importance of distinguishing and tracking what has been happening in different parts of the distribution rather than relying on average or aggregate statistics, with the experience of those towards the top having a major impact on such statistics but not being representative of what is going on across much of the distribution. While some other countries (such as Ireland) have also seen a substantial shift from DB to DC schemes over this period, the implications for the gender pensions gap have not been studied so as well as key to understanding trends in Great Britain these findings are of broader relevance to such contexts, and other countries at an earlier stage on a similar path.

## Decomposing the gender pension wealth gap

We now decompose the gender gap in pension wealth using the Kitagawa/Oaxaca-Blinder decomposition method (Kitagawa, 1955; Oaxaca, 1973; Blinder 1973). This aims to decompose the differences in the expected average outcome $Y$ (pension wealth in our case) between two groups (men and women). If we note the group of men as A and the group of women as B, this difference is: $G=E({Y}^{A})-E({Y}^{B})$. Because of the properties of the linear estimation this gap can be split into two components: the part explained by differences in characteristics or endowments of certain variables and the part due to different coefficients or returns to these characteristics (also called ‘unexplained’ part). This second component reflects different returns to endowments and the interaction between endowments and coefficients.

The twofold decomposition that we apply results from the notion that there is some non-discriminatory coefficients vector ${\beta }^{* }$ that should be used to determine the contribution of the differences in the endowments of predictors. That non-discriminatory vector could be a weighted average of the vector of two groups (where usually half the weight is given to each group) or, as Neumark (1988) or Oaxaca & Ransom (1994) propose, the coefficients of a pooled regression of both groups together (men and women). We will employ this approach and use the pooled regression coefficients as vector ${\beta }^{* }$, adding a group indicator to the estimation as suggested by Jann (2008) to avoid a possible bias towards the explained part.

The outcome difference can then be written as:

$$G=\mathop{\underbrace{{[E({X}^{A})-E({X}^{B})]}^{{\prime} }\,{\beta }^{* }}}\limits_{{Endowments}\,{Part}}+\mathop{\underbrace{[E{({X}^{A})}^{{\prime} }({\beta }^{A}-{\beta }^{* })+E{({X}^{B})}^{{\prime} }({\beta }^{* }-{\beta }^{B})]}}\limits_{{Unexplained}\,{Part}}$$

Where ${X}^{A}$ and ${X}^{B}$ represents the characteristics or ‘endowments’ of each group, and ${\beta }^{A}$ and ${\beta }^{B}$ the coefficients or returns to these endowments for men and women, respectively. We now have a “two-fold” decomposition of the gap $G=Q+U$ where the first component $Q={[E({X}^{A})-E({X}^{B})]}^{{\prime} }{\beta }^{* }$ is the part of the average wealth differential that is “explained” by group differences in the predictors ${X}^{A}$ and ${X}^{B}$ when applied the non-discriminatory coefficients (the “quantity or endowments effect”) and the second summand $U=E{({X}^{A})}^{{\prime} }({\beta }^{A}-{\beta }^{* })+E{({X}^{B})}^{{\prime} }({\beta }^{* }-{\beta }^{B})$ is the “unexplained” part. The latter is sometimes attributed to ‘discrimination’, but we refrain from interpreting it that way because it also captures all potential effects of differences in unobserved variables.

The set of predictors we are able to include using the variables gathered by the WAS is wide-ranging, comprising socioeconomic, labour-market and behavioural variables. The different shares of men and women that have a degree are captured by the “degree qualification” dummy, as is the share of each group that has either a managerial or a high professional job, according to the National Statistics Socio-Economic Classification (NS-SEC) of the ONS. Among the labour market variables, we have included the proportion in each group with a part-time job as the main job, the proportion self-employed, the proportion having spent 2 years or more out of the labour market, and the employment income (employee or self-employed salary). Due to its distinct role in pension entitlement, we have also included a variable indicating working in the Public Sector (central or local government, public company or industry, NHS, armed forces or universities), as well as having a DB rather than DC scheme as main plan. We have also included a set of binary variables related to financial literacy and attitudes. Our ‘pension knowledge’ variable is a dummy with a value one when the respondent affirms to not disagree, agree or strongly agree with the statement “I feel I understand enough about pensions to make decisions about saving for retirement”. The ‘saver attitude’ variable includes those who do not agree, disagree or strongly disagree with the statement “I tend to buy things on credit and pay it off later”, and the risk-taker attitude includes those who, in the statement “If you had a choice between a guaranteed payment of one thousand pounds and a one in five chance of winning ten thousand pounds, which would you choose?” choose the riskier option with a higher expected value over the safer one.

Some of the questions involved appeared only in the WAS questionnaire from the second wave of the survey - crucially, the one recalling the work history and having been out of the labour market for a period - so we have run this analysis on Wave 2 along with the latest available (Round 7). We now limit our sample to individuals between 30 and 65 years old who responded to the questionnaire in person and not by proxy, since only in that case are the financial literacy and attitudes questions asked. Table 5 shows descriptive statistics of the different average ‘endowment’ for the full set of variables analysed for men and women in the for both the start and end of the period. Note for example how the share of women in the sample that have been out of work or that have part-time jobs largely exceeds that of men, whereas the share of men that have ‘pension knowledge’ is larger than that of women. The different distribution (endowment) of these and other variables across genders will explain a good deal of the gap, as we will see later.^11^

**Table 5:** Descriptive statistics of labour market variables by gender in Wave 2 (2008–10) and Round 7 (2018–20) of WAS

	Wave 2 (2008–2010)		Round 7 (2018–2020)	
	Men	Women	Men	Women
Total Private Pension Wealth (£)	117448	72788	164516	102709
Total Private Pension Wealth (IHS transformed)	9.03	8.04	9.99	9.40
Employment Income (£)	23370	19912	25028	20968
Employment Income (IHS transformed)	9.3	9.7	9.4	9.6
Age (Years)	45.3	45.6	45.9	45.8
Pension Knowledge (%)	72.2	56.9	72.8	58.2
Part Time Job (%)	6.2	44.7	8.0	43.0
Saver Attitude (%)	85.4	85.7	85.4	85.9
Risktaker Attitude (%)	25.5	17.9	25.7	17.5
Self-Employed (%)	11.9	6.5	11.5	7.5
Long-term out of work (%)	9.0	32.3	11.1	33.1
Degree Qualification (%)	31.2	28.7	42.2	43.3
Manager/High Professional Job (%)	47.6	43.5	49.3	44.2
DB 0% of Pension Wealth (%)	54.2	47.7	56.3	50.9
DB < 50% of Pension Wealth (%)	5.5	3.0	4.2	3.1
DB 50–75% of Pension Wealth (%)	4.0	2.5	3.8	2.7
DB > 75%of Pension Wealth (%)	36.4	46.8	35.7	43.3
Public Sector Job (%)	21.5	39.2	16.7	34.4

Descriptive statistics for men and women of dependent variable (Total Pension Wealth) and covariates included in the Oaxaca-Blinder decomposition. Income and Wealth measured in 2020 GBP. All other variables represent the prevalence in each group, in percentage. Sample for individuals 30–65 years old with no ‘Pension in Payment’ wealth and who respond to the questionnaire in person, in Wave 2 and Round 7 of the Wealth and Assets Survey in Great Britain

The results of the Oaxaca-Blinder decomposition estimation are presented in Table 6. To address the common issues of right-skewness and zero and negative values in wealth data, we use the Inverse Hyperbolic Sine (IHS) transformation. The decomposition is therefore applied to the difference between men and women in the average of this transformed variable. Note changes in this IHS difference approximate percentage point changes in the relative gap. This happens because the IHS transformation behaves like a logarithm for positive wealth, meaning it captures proportional changes rather than absolute ones, especially across the main part of the distribution rather than the extreme tails. This measure thus reflects relative differences in pension wealth.

**Table 6:** Results for a two-fold Oaxaca-Blinder decomposition (pooled reference group) for Total Pension Wealth by gender

	Wave 2 (2010)			Round 7 (2020)		
VARIABLES	Overall	Explained (‘Endowments’ effect)	Unexplained (‘Coefficients and interaction ‘effect)	Overall	Explained (‘Endowments’ effect)	Unexplained (‘Coefficients and interaction ‘effect)
Pension Knowledge		0.0960***	0.366***		0.0824***	0.116
		(0.0161)	(0.126)		(0.0154)	(0.128)
Part Time		0.259***	−0.132***		0.350***	−0.0837*
		(0.0429)	(0.0429)		(0.0411)	(0.0497)
Saver Attitude		−0.000110	0.117		−0.000778	0.284
		(0.000486)	(0.217)		(0.00153)	(0.220)
Risk Taker Attitude		0.0107	0.100**		0.00545	0.00253
		(0.00818)	(0.0433)		(0.00931)	(0.0476)
Self Employed		0.0577***	0.00538		−0.00594	−0.0214
		(0.0174)	(0.0504)		(0.0158)	(0.0690)
Long_Term_Out_of_Work		0.255***	−0.130***		0.176***	−0.0222
		(0.0288)	(0.0441)		(0.0260)	(0.0466)
Degree_Qualification		0.00577	−0.119*		−0.00497	−0.0929
		(0.00359)	(0.0620)		(0.00598)	(0.0849)
Manager/Professional Job		0.0525***	−0.0725		0.0456***	0.0386
		(0.0152)	(0.0937)		(0.0129)	(0.0917)
DB Share > 0% and <50%		0.146***	−0.0773***		0.0385**	−0.0399***
		(0.0256)	(0.00983)		(0.0165)	(0.00912)
DB Share 50–75%		0.0847***	−0.0579***		0.0397**	−0.0236***
		(0.0231)	(0.00793)		(0.0165)	(0.00839)
DB Share >75%		−0.636***	−0.737***		−0.288***	−0.237***
		(0.0690)	(0.0827)		(0.0491)	(0.0689)
Public_Sector_Job		−0.0301*	0.0119		−0.0634***	0.00177
		(0.0163)	(0.0534)		(0.0157)	(0.0399)
Income		−0.0290***	0.372		−0.0504**	0.169
		(0.0110)	(0.483)		(0.0255)	(0.594)
Age		−0.0344*	2.933***		−0.0121	1.136**
		(0.0183)	(0.446)		(0.0208)	(0.446)
Group_A (Men)	9.033***			9.990***		
	(0.0843)			(0.0838)		
Group_B (Women)	8.042***			9.402***		
	(0.0842)			(0.0772)		
Difference (Gender Pension Wealth Gap)	0.991***			0.588***		
	(0.119)			(0.114)		
Explained Part (“Endowments”)	0.237**	% Explained by endowments	23.92%	0.312***	% Explained by endowments	53.06%
	(0.0969)			(0.0890)		
Unexplained Part (“Coefficients + Interaction)	0.754***	% Explained by returns	76.08%	0.276***	% Explained by returns	46.94%
	(0.102)			(0.0981)		
Constant			−1.826**			−0.951
			(0.740)			(0.859)
Observations	9897			8858		
*** p < 0.01, ** p < 0.05, * p < 0.1						

Sample of individuals 30–65 years old with no ‘Pension in Payment’ wealth and who respond to the questionnaire in person, in Wave 2 and Round 7 of the WAS in Great Britain. Dependent variable Total Pension Wealth Gap (Inverse Hyperbolic Sine (IHS) Transformed). All variables are categorical dummies except age and Income (Employment Adjusted Income with IHS transformation). Omitted category for DB share is DB Share = 0%. Note that *** indicates statistical significance at the 1% level, ** at the 5% level, and * at the 10% level

Our decomposition results show a significant narrowing trend of the gender wealth gap, from 0.991 in 2010 to 0.588 in 2020. In untransformed data (Table 3) we already observed that while the absolute gender pension wealth gap actually grew between 2008 (£87k) and 2020 (£120k), the standard relative gap (with men’s value as baseline) slightly decreased, from 57.0% to 52.7%.^12^

The decomposition analysis (Table 6) of the gender gap in the IHS transformed pension wealth variable shows that a key factor for this convergence was a large fall in the ‘unexplained’ part of the gap (from 0.754 in 2010 to 0.276 in 2020). This suggests that the differences in the pension wealth returns men and women received for similar characteristics decreased over the decade. On the other hand, the ‘explained’ part of the gap grew from 0.237 to 0.312. As a result, by 2020, differences due to observable characteristics became the main reason (accounting for 53%) for the remaining IHS gap. In other words, while returns became more equal, differences in underlying characteristics became the primary source of the persistent relative disadvantage for women.

Looking at specific factors, the decomposition offers further insights. For example, differences in returns related to age contributed significantly to the unexplained gap in both periods (widening it), though less so in 2020 than in 2010. Pension Knowledge, which descriptive statistics show is consistently higher among men (Table 5), generated differences in returns that widened the unexplained gap in 2010, but this effect disappeared by 2020. The fact that men had higher knowledge levels, however, consistently explained a small part of the gap due to endowments.

Some factors show little impact on the gap: Saver Attitude is very similar between genders (see Table 5) and had negligible effects. Men report a higher Risktaker Attitude, but this does not translate into a significant explained. Degree Qualification, where women closed the gap and slightly surpassed men by 2020 (Table 5), also had minimal impact in explaining the IHS mean gap (Table 6).

The most powerful explanations for the IHS-scaled gap, however, lie in differences related to labour market experiences and pension plan types. Diverging Labour Market participation patterns remain key drivers of the ‘explained’ gap. Women’s much higher rates of Part-Time work clearly translate into a large, positive contribution to the explained gap in both 2010 (0.259) and 2020 (0.350). This directly reflects the descriptive data (Table 5), where 43–45% of women worked part-time versus only 6–8% of men. Similarly, women’s higher likelihood of Long-Term absence from the workforce (around 32–33% versus 9–11% for men) explained another large part of the gap in both years (0.255 in 2010; 0.176 in 2020). These findings highlight the lasting pension penalties linked to employment patterns linked to parenthood and more common among women, showing how career breaks and part-time work create significant gender gaps in relative pension wealth.

In contrast, having higher shares of pension wealth in DB plans significantly *reduced* the relative gender gap in both periods, mainly through the endowment component linked to having over 75% in DB (Table 6: explained effect of −0.636 in 2010; −0.288 in 2020). This aligns with the descriptive statistics (Table 5) showing more women in this high-DB category (43–47% vs 36% for men). This difference is likely connected to women’s higher employment rates in the Public Sector (Table 5), where DB schemes have been historically prevalent. Therefore, women’s greater concentration in high-DB plans helped mitigate the overall relative gap. However, the analysis also indicates that this mitigating effect had lessened somewhat by 2020.^13^

To ensure the robustness of these findings against the influence of the wealth distribution’s skewness, we also performed a decomposition at the median using Re-centered Influence Function (RIF) regression (Firpo et al., 2009; Rios-Avila, 2020). The results, presented in Appendix E (Table 11), are qualitatively consistent with our main analysis.^14^ They confirm that even for the ‘typical’ individual at the median of the distribution, differences in labour market characteristics—specifically part-time work and career gaps—remain the primary drivers of the explained gender pension gap, as well as does having a higher share of pension wealth in DC schemes. This reinforces the conclusion that structural labour market inequalities, even if greater in absolute terms as one goes up the distribution, are central to explaining the gender disparity across the board.

The only similar decomposition analysis we know of is that by Meriküll et al. (2021) with register data for Estonia, which covered overall wealth and its different components including pension wealth. With a different set of characteristics, they also found an unexplained gap for private pensions in favour of men. Among the variables, they also find risk aversion differences do not reduce the unexplained part significantly. The detailed analysis of labour market path characteristics we undertake here -career breaks, part-time work- is not available in their administrative datasets. Nor is, of course, the structural aspects of the British system -particularly the impact of different types of pension plans we have foregrounded here.

Overall, our results underline the importance of structural factors in the broader context of pension system change in driving the observed pension wealth gaps, with only a very limited role for individual differences in relevant knowledge and attitudes to risk. In a system where occupational pensions play such a dominant role, experience in the labour market is bound to be key for individuals. However, the nature of those pensions is also key: the ongoing decline in the role of traditional DB schemes and the corresponding rise in Defined Contribution (DC) plans, as explored in Section 5, has major consequences for gender equality in pensions. As the influence of DB schemes wanes, a factor that narrowed the relative pension gap is diminishing. At the same time, the growth of DC plans, where outcomes are more closely tied to individual contributions, means gender wage gaps and occupational segregation by gender, have greater importance, and the penalty for women labour market paths is larger.

Note that, while DB plans have linear formulas to accumulate pension rights proportionally to income and seniority, DC plans are more directly linked to *exponential* returns of accumulated funds and tend to amplify the effects of the lower contribution of part-time workers and the pension penalty for career breaks, when the contribution to the plan is stopped. This creates even greater hurdles for achieving gender parity in pension wealth in retirement, with policy implications to which we return in our concluding discussion below.

## Discussion and conclusions

Wealth is often studied at the household level but individual wealth data collected for Great Britain in the Wealth and Assets survey provides us with an exceptional opportunity to assess the gender wealth gap and its evolution over time, with a particular emphasis on the role of private pension wealth, which is exceptionally important in the British case compared to other European countries. We found that gender wealth gaps are much more substantial when private pension wealth is included than when – as is more often the case – they are left out. In the 2018–20 wave of WAS the gap between mean wealth for women versus men was 34.0% (of the men’s mean) when pension wealth was included versus 16.3% when it was not. This gender wealth gap including private pensions was more pronounced among men and women living in couples than among single individuals; among couples both wealth levels and the gender gap were seen to increase with age. Apart from pension wealth, the other substantial contributor to the overall wealth gap was financial and business wealth; main residence wealth by contrast showed a modest gap in favour of women.

We also found that the average gap for private pension wealth, while showing a steady trend in relative terms, has increased markedly in real absolute terms since 2008, as have the gap for financial and business wealth and the overall wealth gap. The pension wealth gap in Great Britain was seen to be very pronounced towards the top of the distribution, where it has also increased markedly over the period covered. There was also a substantial increase in the gender gap for financial and business wealth at the higher deciles of the distribution.

In-depth analysis of the gender gap in private pension wealth for working age individuals brought out the importance of the value of Defined Contribution (DC) as opposed to Defined Benefit (DB) schemes in understanding patterns of change over time – that is, schemes where the individual relies on returns from their accumulated pension contributions rather than simply based on their wage when in work and years of service. The proportion of pension wealth in DC schemes has increased over time, and in parallel the gender gap in this form of pension wealth has increased markedly towards the top of the wealth distribution. Decomposition analysis applied to that gap revealed that the difference in characteristics (“endowments”) between men and women captured in the model accounted for about 53% of the private pension wealth gap between men and women in 2018–20, a larger share than a decade earlier. Differences in employment income were a strong and significant contributor to the gap, as were other labour market variables related to care and parenting that show difference prevalence by gender: the higher share of women in part time jobs and taking long periods out of work. The lower share of women in managerial and high-technical jobs also contributed to the gap. On the other hand, the share of women in public sector jobs and in jobs with DB instead of DC pension plans served to reduce the gender pension wealth gap. While attitudes to saving and risk-taking did not seem to have a significant impact on the gap in the pension wealth gap, differences in knowledge about ‘making decisions about saving for retirement’ did make some contribution.

Overall, these results underline the importance of structural factors in the broader context of pension system change in driving the observed pension wealth gaps in Great Britain and, potentially, in other countries moving their pension system in that direction. In a system where occupational and private pensions play such a dominant role, experience in the labour market is fundamental for individuals while the nature of those pensions and how it is changing is also key. The waning influence of DB schemes means that a factor that serves to narrow the relative pension gap is diminishing. At the same time, the growth of DC plans where outcomes are more closely tied to individual contributions means gender wage gaps and occupational segregation by gender, together with career interruptions and greater part-time working for women, all become more important in driving outcomes in terms of who has effective access to occupational/private pensions and the generosity of the pension for those who are covered. As pointed out before, the exponential nature of the building of wealth over DC plans, where the market value evolution of pension savings is practically the sole determinant of future pension rights, tends to amplify baseline differences in contributions and the effect of career breaks and contribution gaps. Even more, given the nature of the compounds returns effect, if these occurs at the early stages of the career life, when mothers tend to take more breaks and reduced working hours than parents (Fernández-Kranz & Rodríguez-Planas, 2011; Kleven, Landais & Søgaard, 2019).

This can be seen as creating even greater hurdles for achieving gender parity in pension wealth in retirement, with implications for both pension policy and labour market policies in Great Britain and more broadly. From the perspective of the overall pension structure in place in Great Britain, the state pension based on social security contributions and means-tested Universal Credit represent the sole pension income for workers who fail to achieve coverage by occupational schemes, with very few low-paid workers being able to save into purely private pensions. Tax incentives to support such savings will continue to benefit the higher-paid disproportionately, so the level of support provided by the social security-based state pension will continue to be a central plank in underpinning living standards in retirement for lower-paid and marginal workers. This does not take away from the role of policies to broaden the coverage of occupational schemes by measures such as ‘nudges by making the default choice ‘opting in’ rather than ‘opting out’ of such schemes. In terms of structural change, it is now very difficult for policy to reduce the momentum behind the on-going switch from Defined Benefit to Defined Contribution schemes, where action at a much earlier point might have had much more impact. However, continued support of existing Defined Benefit schemes in the public sector is within the scope of government action, resisting the temptation to follow the private sector especially in public sector organisations that are at some distance from the central civil service in terms of governance, terms and conditions.

Other countries may well draw lessons from the UK experience, notably from how difficult it has been for policy there to intervene effectively, in a system where non-state provision dominates, to avoid or offset changes in the way occupational and private pensions operate over recent decades that have been detrimental from the perspective of pension gender wealth gaps, and indeed more broadly in terms of pension security for workers in retirement. This is despite the very substantial expenditures to support that non-state provision through tax breaks, which have created a variety of hard-to-solve problems in themselves in terms of rigidities and perverse incentives.

Pension structures and policies are only part of the story, in Great Britain and elsewhere, with broader labour market policies also central to addressing differences between men and women in pension entitlement and pension wealth. While there has been some progress over time in reducing gender pay gaps in many countries more remains to be done, and the fact that career interruptions and part-time working are much more for women, alongside occupational segregation by gender, all underpin the poorer pension provision available to women. These are all complex phenomena but potentially can be influenced by the full range of labour market policies promoted for example by the EU’s employment targets and associated national plans; the reduction in the labour market disadvantage of women on average is key to promotion of pension equality.

A final issue to highlight of major policy relevance is the implications of increasing reliance on Defined Contribution schemes for the vulnerability of the system, and those depending on it, to financial shocks. The potential consequences of a severe downturn in financial markets, much of crisis and market failure, are exacerbated by this shift. The ECB has pointed not only to it making retirement savings more vulnerable to shocks, but also affecting the demand for different asset classes by pension funds, lowering the demand for long-duration bonds while increasing it for equities – potentially contributing to households’ retirement savings becoming more uncertain and retirement income being more unequally distributed (Rousová et al., 2021).

In concluding it should be noted that the data employed here for Great Britain from the Wealth and Assets Survey offer significant potential for further research. In particular, the fact that wealth has been also measured longitudinally at individual adult level over a significant period means that the dynamics of wealth can be investigated. This would include comparing what happens to the wealth of men versus women by type of wealth over the life-cycle and through various labour market ‘events’ such as moving from full-time to part-time work, experiencing unemployment or career interruption. The extent of attrition in the survey over time and the possible scarcity of observations for particular events, however, means that considerable care will have to be exercised in exploiting this dynamic potential. More broadly, the integration of pension wealth into studies of wealth inequality, ideally incorporating both public and private pensions, is a clear priority if the overall distribution of wealth and the drivers of differences across countries and change over time are to be properly understood.

## Figures and Tables

**Fig. 7:**
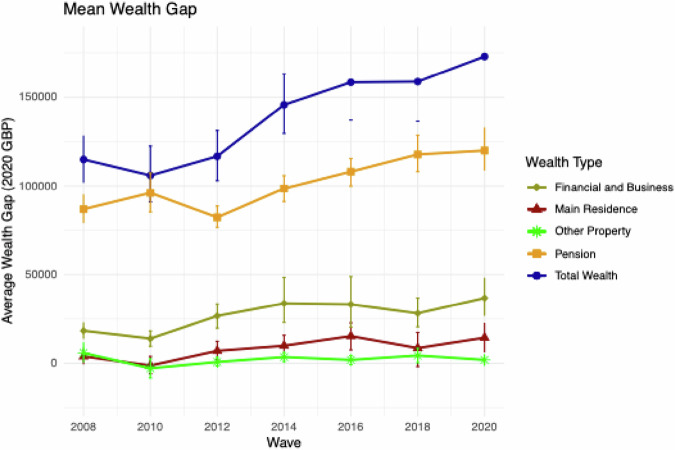
**Gender wealth gap in total wealth (including pension wealth) and in each main wealth component, WAS 2006–08 to 2018–20**. Standard errors obtained by bootstrap (n = 500). Other Property includes all property (other than the main residence) and physical wealth (valuables, household contents, cars, etc). Main Residence wealth in couples not split (assigned fully to both members of the couple)

**Fig. 8:**
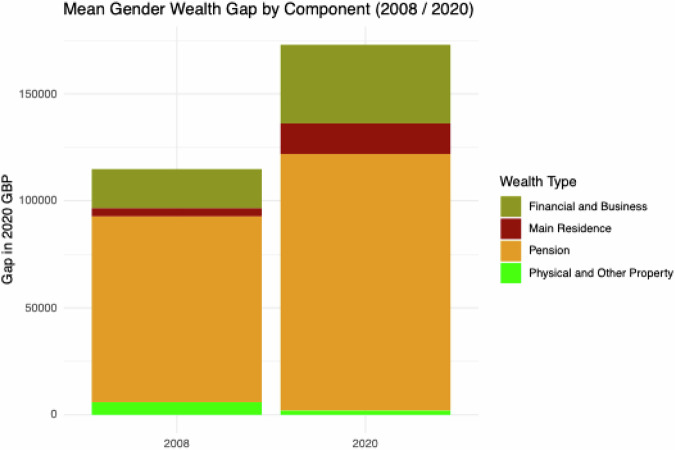
**Contribution of each wealth component to the Gender gap in total wealth in the first and latest available waves (2006–08 and 2018–20). Main Residence Wealth not split**. Average gender wealth gap (aggregate total and coloured by components) for all population of 30 years of age and older for Wave 1 (2006–2008) and Round 7 (2018–2020) of the Wealth and Assets Survey. Values in absolute real GBP (2020 prices)

**Fig. 9:**
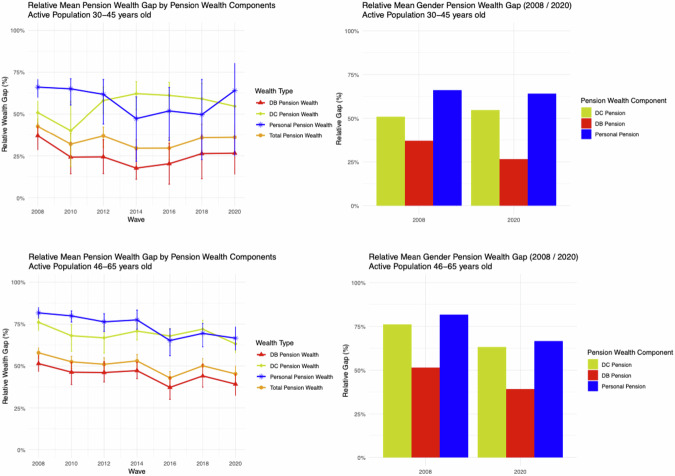
**Evolution of relative pension wealth gap for active population (age groups 30–45 and 46–65)**. Evolution of relative mean pension wealth for total pension wealth and for each pension wealth component across the seven waves of WAS (2008–2020) in the left part of the graph. Top-left shows this evolution through all the seven waves for the 30–45 age group, while bottom left shows the evolution for the 46–65 age group. The right-hand side bar-graphs show relative gap in each component in the total pension wealth gap (except spouse pension wealth) in 2008 and 2020, for the 30–45 age group (top right) and for the 46–65 active age group (bottom right)

## Data Availability

Data used for this research (Wealth and Assets Survey) can be obtained from the Office for National Statistics (United Kindgom) via the UK Data Service. Code used for the analysis of the data is available upon request.

## Appendix

*A. The influence of main residence wealth split vs considering it as a public good*.

We calculate the gender total wealth gap for all waves considering the full value of the main residence for both components of the couple (no split), thus assuming the value of the house as a full public good for household members. This increases slightly the total gap, but no major changes are seen in the trend across time (Fig. 7).

The increase is due to the reduction of the average main residence gap in favour of women when comparing this to our baseline estimation (main residence split equally), as can be seen in Table 7 and Fig. 8. Note that not splitting implies overweighting couples in the sample, which are given in aggregate more wealth than it really exist. Given that part of the main residence gap in favour of women occurred among single headed households, this ‘reweighting’ gives this group less importance, hence the disappearance of the main residence gap favouring women (there is now a gap favouring men) and the resulting increase in the overall total wealth gap favouring men.

**Table 7:** Mean gender wealth Gap in total wealth and in each main wealth component, WAS 2006–08 and 2018–20

All Pop. Over 30						
Wealth Component	Gap (£)	Contribution to Total Wealth Gap (%)	Gap (£)	Contribution to Total Wealth Gap (%)	Change in Gap (£)	Contribution to Change of Total Gap (%)
	Wave 1 (2006–2008)		Round 7 (2018–2020)		Change between Wave 1 and Round 7	
Financial and Business Wealth	18291	16%	36722	21%	18431	32%
Main Residence Wealth	3902	3%	14367	8%	10465	18%
Other Property Wealth	5822	5%	1962	1%	−3860	−7%
Pension Wealth	86853	76%	119921	69%	33068	57%
Total Wealth	114867	100%	172972	100%	58105	100%

Average gender wealth gap and contribution to total wealth gap for each wealth component. All population of 30 years of age and older. Wave 1 (2006–2008) and Round 7 (2018–2020) of the Wealth and Assets Survey. Main Residence wealth in couples not split (assigned fully to both members of the couple)

*B. The influence of widowhood in the estimates of the gender wealth gap*.

In order to assess whether a different prevalence of widowhood by gender could have an impact on the gender wealth gap, we have re-estimated the gap by age groups excluding widowed individuals.

We can see that compared to the original baseline data, Appendix Table 8 shows that the levels of wealth for both men and women are just slightly lower and ‘the gap both at the mean and at the median slightly higher in both absolute and relative terms. While the gender gap does not change for couples it does increase significantly for single non-cohabiting individuals once we exclude widowed individuals. When comparing to the baseline results in Table 1 in the main text we can see that the increase is marked both at the mean and at the median and both in absolute and relative terms.

**Table 8:** Wealth Levels and Gender Gaps, Great Britain, 2018–20

*1a. Wealth levels and wealth gap by gender and couple status including pension wealth*										
		Wealth Levels					Wealth Gaps: Absolute (£ x 1000) and Relative (% of men’s wealth)			
		Mean (£ x 1000)		Median (£ x 1000)			Mean		Median	
		Estimate	S.E.	Estimate	S.E.		Estimate	S.E.	Estimate	S.E.
All	Men	466	10	241	5	Gap	172	11	73	172
	Women	294	4	168	3		37.0%	***	30.4%	37.0%
Couple	Men	502	12	273	7	Gap	201	13	87	201
	Women	302	5	186	4		39.9%	***	31.9%	39.9%
Single	Men	349	14	158	10	Gap	75	16	54	75
	Women	274	9	104	7		21.4%	***	34.3%	21.4%

*1b. Wealth levels and wealth gap by gender and couple status excluding pension wealth*										
		Wealth Levels					Wealth Gaps: Absolute (£ x 1000) and Relative (% of men’s wealth)			
		Mean (£ x 1000)		Median (£ x 1000)			Mean		Median	
		Estimate	S.E.	Estimate	S.E.		Estimate	S.E.	Estimate	S.E.
All	Men	236	7	133	2	Gap	46	7	16	3
	Women	190	3	117	2		19.6%	***	12.4%	***
Couple	Men	249	9	141	2	Gap	51	9	11	3
	Women	198	3	130	3		20.4%	***	7.6%	***
Single	Men	194	8	83	9	Gap	26	9	32	10
	Women	168	6	52	6		13.5%	***	38.2%	***

Estimation of individual total net wealth and individual total net wealth excluding pension wealth for the population over 30 years of age. Singlehood refers to single, separated and divorced individuals not currently living with a partner. **Widowed individuals are excluded from the sample**. Data from Round 7 of Wealth and Assets Survey (collected 2018–2020). Standard Errors calculated via bootstrap (standard deviation of bootstrapped samples). Significance of the gap based on the distribution of the bootstrap estimates and using the t-statistic, *** indicates statistical significance at the 1% level, ** at the 5% level, and * at the 10% level

This robustness check reveals that there is a slight downward bias in the baseline gender wealth gap estimates due to the composition effect of widowhood favouring women in the single-living households. That is, the gap would be larger if widowed individuals were excluded from the sample. However, we have decided to keep the baseline gap including all individuals, for our aim is to first depict the gender wealth gap in the most comprehensive way. In the specific analysis of the pension wealth gap, which takes into consideration mainly labour market variables, we have then excluded pensions obtained from a deceased spouse.

*C. Evolution and Composition of the Relative Gap*

*D. Non-response by gender in key covariates*

We have checked item non-response (NA values) for men and women respondents in key values of covariates used in the decomposition.

**Table 9:** Missing values for key variables by gender (% of the 30–65 years old active population sample)

	Wave 2 (2008–2010)		Round 7 (2018–2020)	
	Men	Women	Men	Women
Pension Wealth	0.00	0.00	0.00	0.00
Employment Income	0.00	0.00	0.00	0.00
Age	0.00	0.00	0.00	0.00
Pension Knowledge	0.04	0.03	2.73	3.29
Part Time Job	0.00	0.00	0.00	0.00
Saver Attitude	1.01	2.01	0.48	0.37
Risktaker Attitude	2.06	2.06	0.81	0.91
Self-Employed	0.00	0.00	0.00	0.00
Long-term out of work	11.96	14.31	1.36	2.79
Degree Qualification	0.00	0.00	0.00	0.00
Manager/High Professional Job	0.00	0.00	0.00	0.00
Defined Benefit Plan Wealth	0.00	0.00	0.00	0.00
Public Sector Job (%)	21.20	34.56	19.46	28.83

*E. Alternative decomposition analysis*

**Table 10:** Results for a two-fold Oaxaca-Blinder decomposition for Total Pension Wealth by gender, excluding public-sector question to avoid selection bias due to differential rate of missing by gender

	Wave 2 (2010)			Round 7 (2020)		
VARIABLES	Overall	Explained (‘Endowments’ effect)	Unexplained (‘Coefficients and interaction ‘effect)	Overall	Explained (‘Endowments’ effect)	Unexplained (‘Coefficients and interaction ‘effect)
Pension Knowledge		0.0938***	0.343***		0.0988***	0.180
		(0.0132)	(0.105)		(0.0151)	(0.116)
Part Time		0.164***	−0.0917**		0.254***	−0.0764
		(0.0341)	(0.0385)		(0.0367)	(0.0474)
Saver Attitude		0.000284	−0.114		−0.00262	0.315
		(0.000832)	(0.192)		(0.00267)	(0.204)
Risk Taker Attitude		0.0113	0.0967***		0.0119	−0.00452
		(0.00701)	(0.0363)		(0.00820)	(0.0426)
Self Employed		0.115***	−0.0191		0.0195*	−0.0292
		(0.0152)	(0.0313)		(0.0114)	(0.0418)
Long_Term_Out_of_Work		0.208***	−0.156***		0.199***	−0.0464
		(0.0241)	(0.0547)		(0.0255)	(0.0622)
Degree_Qualification		0.00711**	−0.131**		−0.00843	−0.108
		(0.00346)	(0.0509)		(0.00681)	(0.0755)
Manager/Professional Job		0.0679***	0.0395		0.0472***	0.0379
		(0.0135)	(0.0747)		(0.0130)	(0.0831)
DB Share > 0% and <50%		0.141***	−0.0655***		0.0500***	−0.0386***
		(0.0223)	(0.00810)		(0.0160)	(0.00835)
DB Share 50–75%		0.0876***	−0.0518***		0.0486***	−0.0251***
		(0.0199)	(0.00645)		(0.0156)	(0.00764)
DB Share >75%		−0.327***	−0.607***		−0.247***	−0.271***
		(0.0605)	(0.0575)		(0.0490)	(0.0572)
Income		0.114***	0.103		0.154***	−0.0109
		(0.0194)	(0.163)		(0.0360)	(0.234)
Age		−0.0190*	3.272***		0.0242	1.177***
		(0.0104)	(0.380)		(0.0171)	(0.420)
Group_A (Men)	8.222***			9.357***		
	(0.0799)			(0.0845)		
Group_B (Women)	6.599***			8.374***		
	(0.0734)			(0.0773)		
Difference (Gender Pension Wealth Gap)	1.623***			0.983***		
	(0.109)			(0.115)		
Explained Part (“Endowments”)	0.664***			0.649***		
	(0.0887)			(0.0916)		
Unexplained Part (“Coefficients + Interaction)	0.959***			0.333***		
	(0.0874)			(0.0913)		
Constant			−1.659***			−0.766
			(0.495)			(0.573)
Observations	13544			11156		
*** p < 0.01, ** p < 0.05, * p < 0.1						

Sample of individuals 30–65 years old with no ‘Pension in Payment’ wealth and who respond to the questionnaire in person, in Wave 2 and Round 7 of the WAS in Great Britain. Dependent variable Total Pension Wealth Gap (Inverse Hyperbolic Sine (IHS) Transformed). All variables are categorical dummies except age and Income (Employment Adjusted Income with IHS transformation). Omitted category for DB share is DB Share = 0%. Note that *** indicates statistical significance at the 1% level, ** at the 5% level, and * at the 10% level. Sample is larger than main text analysis because Public Sector covariate (included in the baseline model but not here) had as missing all unemployed individuals (was only asked to those working at the moment)

**Table 11:** Results for a two-fold Oaxaca-Blinder decomposition for Total Pension Wealth by gender estimation at the median using RIF-REGRESSION on the original variable

	Wave 2 (2010)			Round 7 (2020)		
VARIABLES	Overall	Explained (‘Endowments’ effect)	Unexplained (‘Coefficients and interaction ‘effect)	Overall	Explained (‘Endowments’ effect)	Unexplained (‘Coefficients and interaction ‘effect)
Pension Knowledge		564.6***	3210***		677.9***	2050
		(115.5)	(932.8)		(184.6)	(1622)
Part Time		2307***	−404.5		2164***	−595.0
		(320.0)	(294.7)		(471.4)	(485.7)
Saver Attitude		−4.746	67.99		−9.727	674.7
		(13.86)	(1625)		(19.07)	(2722)
Risk Taker Attitude		40.35	152.3		153.1	−36.00
		(60.36)	(326.5)		(119.5)	(609.9)
Self Employed		548.1***	−426.2		409.3***	−417.3
		(129.8)	(348.3)		(153.9)	(602.6)
Long_Term_Out_of_Work		1426***	−1159***		2840***	−560.8
		(201.7)	(307.1)		(327.2)	(541.2)
Degree_Qualification		28.16	−202.7		−56.43	341.7
		(23.37)	(470.3)		(68.18)	(1080)
Manager/Professional Job		396.9***	−1296*		854.2***	−1577
		(115.1)	(714.9)		(233.3)	(1269)
DB Share > 0% and <50%		1113***	−394.5***		530.1**	−446.2***
		(196.4)	(106.8)		(227.7)	(164.1)
DB Share 50–75%		620.9***	−364.0***		561.8**	−164.9
		(169.7)	(83.63)		(235.4)	(185.7)
DB Share >75%		−4356***	−3351***		−3824***	−3096***
		(474.4)	(683.1)		(654.1)	(1093)
Public_Sector_Job		−315.9**	−599.7		−1048***	−730.4
		(134.0)	(440.7)		(258.3)	(675.2)
Income		−211.2***	−4108		−367.3*	3539
		(79.18)	(3435)		(188.0)	(5311)
Age		−279.4*	13146***		−203.4	13603**
		(148.1)	(3377)		(349.6)	(5707)
Group_A (Men)	24031***			33428***		
	(611.0)			(1041)		
Group_B (Women)	17570***			24020***		
	(610.6)			(989.6)		
Difference (Gender Pension Wealth Gap)	6460***			9408***		
	(863.8)			(1436)		
Explained Part (“Endowments”)	1878***	% Explained by endowments	29.07%	2681**	% Explained by endowments	28.50%
	(693.6)			(1089)		
Unexplained Part (“Coefficients + Interaction)	4582***	% Explained by returns	70.93%	6727***	% Explained by returns	71.50%
	(740.0)			(1254)		
Constant	313.2					−5859
	(5445)					(8649)
Observations	9897			8858		
*** p < 0.01, ** p < 0.05, * p < 0.1						

Sample of individuals 30–65 years old with no ‘Pension in Payment’ wealth and who respond to the questionnaire in person, in Wave 2 and Round 7 of the WAS in Great Britain. Dependent variable Total Pension Wealth Gap. All variables are categorical dummies except age and Income (Employment Adjusted Income). Omitted category for DB share is DB Share = 0%. Note that *** indicates statistical significance at the 1% level, ** at the 5% level, and * at the 10% level

## Footnotes

1 The WAS measure of pension wealth includes the value of all future pension rights (personal and occupational plans) except for those of the public pension system. Note that in the case of Great Britain, the importance of the public pension system is much lower than the OECD average, as we discuss below.

2 The ONS changed the timeline of the response collection period to match financial years in 2016. Since then waves are called ‘rounds’, the terminology we use for Round 6 (2016-2018) and 7 (2018-2020). See https://www.ons.gov.uk/peoplepopulationandcommunity/personalandhouseholdfinances/incomeandwealth/methodologies/movingthewealthandassetssurveyontoafinancialyearsbasis.

3 Note that, for brevity, we refer to waves by the latest year of the fieldwork in the text and plots (e.g., Wave 1 is plotted in the year 2008 and Round 7 in year 2020).

4 On the basis of the distribution of households by National Statistics Socio-economic classification (NS-SEC) group and the proportion without a car in the area, those more likely to be wealthy were flagged, and in W1 addresses from wealthier postcodes were 2.5 or 3 times more likely to be sampled.

5 In a robustness check in Appendix A we have instead assumed that both partners in a couple each benefit fully from the amenity value of the main residence, treating it in this sense as a public good and attributing the full value to both (as is done for instance in Poterba et al., 2018).

6 Riphahn and Serfling’s (2022) study based on the German Socio-Economic Panel suggested that the gender of the interviewer rather than the respondent had more significant effects on item non-response for wealth-related questions, which was higher for female interviewers. Tranfaglia et al. (2024) found in a survey experiment with the 2021 Survey of Household Economics and Decisionmaking that women’s greater propensity to respond “don’t know” is a major contributor to the measured gender gap in financial literacy, but this relates to general finance-focused questions rather than ones about the respondent’s own finances.

7 For the “Pensions in Payment” component the ONS does not provide information as whether the value was built with a DC or a DB scheme. Our analysis of the pension wealth gap by its components is then run only for the working age population (see Section 5) who are not yet retired, and do not receive any pension in payment. Also note that -as far as we know- other surveys such as HFCS do not generally include pensions in payment when computing pension values.

8 Parenthood has been linked to gender disadvantages for wealth accumulation (Lersch, Jacob, & Hank 2017). In particular, single parenthood (Morelli et al., 2022), which is much more prevalent for women. These studies focus on households in which children are still living in the household. The influence of parenthood in pension wealth, however, is likely to largely reflect later in life even when children have left the parental home. Unfortunately, our dataset does not provide information about parenthood for households in which the child has already left the home (i.e., we cannot know whether the individual is a parent if the child is no longer living in the same household). This has prevented us from using the ‘parenthood variable’ directly in our analysis. Note, however, that implicit career patterns linked to parenthood and more prevalent among women (career breaks, part-time work), will be key in explaining the pension wealth gap (see Section 6).

9 The IFS has recently released a critical report on a change in the valuation wealth -and particularly pension wealth- that the ONS has done from 2022 in the Wealth and Assets Survey (Adam et al., 2025). This change, however, does not affect the waves and rounds used in our analysis.

10 We do not split working age population in two age groups here in order to have enough observations to estimate quantiles with enough precision.

11 We show the share of item non-response (missing values) for all the covariates used in the decomposition analysis in Appendix C. We see there is not a general bias in item non-response by gender (See Appendix Table 9). We observe that there are no missing values in most variables, and in those where there are some (particularly for having been a long period out of the labour market) the rate of non-response is similar for both genders. There is, though, a difference in the rate of missing observations in the ‘works in the public sector’ variable that we use, in which women have a higher rate in both waves analysed (Wave 2 and Round 7). This is due to this question being asked only for working population, so the gender difference in unemployment rate is indirectly causing this bias. For robustness, we have then repeated our decomposition exercise excluding this variable from the regression -therefore including both working and currently unemployed population- and the output is shown in Table 10 in the Appendix. Reassuringly, main results (sign and importance) of the rest of the variables aligns with the full specification decomposition.

12 Note the relative reduction shown by the IHS gap measure (around 41% decrease) is much larger than the reduction shown by the standard relative gap (7.5% decrease). This difference arises from how each measure handles the top end of the wealth distribution. The widening absolute gap suggests large wealth changes concentrate at the top of the distribution, as we have seen in the analysis across the distribution in Section 4.3. The standard relative gap calculation is sensitive to these high values through the average wealth figures, while the IHS transformation compresses them. As a result, the IHS gap is less affected by widening absolute differences at the very top and reflects the change happening across the broader distribution.

13 Note that referring to “differences in returns” -driven by the coefficient and the interaction- may give the impression that these are driven primarily by investment choices made by the individual. In reality, the nature of the scheme involved will most often be the key influence.

14 Note that we have not IHS transformed the quantitative variables (pension wealth gap and income) in the median decomposition, as the median is not sensitive to skewedness as the mean is. Results do not qualitatively change in the estimation using the transformed variable.
